# Spatial transcriptomic characterization of pathologic niches in IPF

**DOI:** 10.1126/sciadv.adl5473

**Published:** 2024-08-09

**Authors:** Christoph H. Mayr, Diana Santacruz, Sebastian Jarosch, Marina Bleck, John Dalton, Angela McNabola, Charlotte Lempp, Lavinia Neubert, Berenice Rath, Jan C. Kamp, Danny Jonigk, Mark Kühnel, Holger Schlüter, Alexander Klimowicz, Jonas Doerr, Alec Dick, Fidel Ramirez, Matthew J. Thomas

**Affiliations:** ^1^ Boehringer Ingelheim Pharma GmbH & Co. KG, Department Immunology and Respiratory Disease research, Birkendorfer Straße 65, 88397 Biberach an der Riß, Germany.; ^2^ Boehringer Ingelheim Pharma GmbH & Co. KG, Global Computational Biology and Digital Sciences, Birkendorfer Straße 65, 88397 Biberach an der Riß, Germany.; ^3^ Boehringer Ingelheim Pharma GmbH & Co. KG, Department Drug Discovery Sciences, Birkendorfer Straße 65, 88397 Biberach an der Riß, Germany.; ^4^ Boehringer Ingelheim Pharmaceuticals Inc., Department of Immunology and Respiratory Diseases Research, 900 Ridgebury Road, Ridgefield, CT 06877 USA.; ^5^ Biomedical Research in Endstage and Obstructive Lung Disease Hannover (BREATH), German Center for Lung Research (DZL), Hannover, Germany.; ^6^ Institute of Pathology, Hannover Medical School, Hannover, Germany.; ^7^ Department of Respiratory Medicine and Infectious Diseases, Hannover Medical School, Hannover, Germany.; ^8^ Institute of Pathology, University Medical Center RWTH University of Aachen, Aachen, Germany.

## Abstract

Despite advancements in antifibrotic therapy, idiopathic pulmonary fibrosis (IPF) remains a medical condition with unmet needs. Single-cell RNA sequencing (scRNA-seq) has enhanced our understanding of IPF but lacks the cellular tissue context and gene expression localization that spatial transcriptomics provides. To bridge this gap, we profiled IPF and control patient lung tissue using spatial transcriptomics, integrating the data with an IPF scRNA-seq atlas. We identified three disease-associated niches with unique cellular compositions and localizations. These include a fibrotic niche, consisting of myofibroblasts and aberrant basaloid cells, located around airways and adjacent to an airway macrophage niche in the lumen, containing SPP1^+^ macrophages. In addition, we identified an immune niche, characterized by distinct lymphoid cell foci in fibrotic tissue, surrounded by remodeled endothelial vessels. This spatial characterization of IPF niches will facilitate the identification of drug targets that disrupt disease-driving niches and aid in the development of disease relevant in vitro models.

## Teaser

Spatial transcriptomics localizes pathologic cell types and identifies cellular niches in IPF lung tissue.

## INTRODUCTION

Recent studies have leveraged the power of single-cell RNA sequencing (scRNA-seq) to provide a cell type–specific perspective on pathological changes in lung diseases, the third leading cause of human mortality (*1*–*3*). These scRNA-seq studies have identified previously unknown fibrosis-specific cell types and molecular changes associated with idiopathic pulmonary fibrosis (IPF), laying the foundation for a new understanding of fundamental biological processes occurring in the diseased fibrotic lung (*4*–*9*). Despite the availability of two antifibrotic therapies for IPF (*10*, *11*), there remains a profound unmet medical need for therapies that address previously unknown pathobiology beyond myofibroblast activity.

The position of a cell, its surrounding neighbors, and tissue structures, such as honeycomb morphology or fibroblastic foci in IPF, are crucial in defining cellular phenotype, disease state, and ultimately cell function and communication (*12*, *13*). However, during scRNA-seq sampling, the original tissue structure is inevitably destroyed, and the single cells lose their spatial location information (*14*). In addition, the biased mechanical extraction process can result in the loss of more fragile cell types such as alveolar type 1 (AT1) cells, distorting cell population distribution (*15*). Thus, an unbiased understanding that goes beyond single-marker imaging, of where fibrotic cell types such as myofibroblasts, *Krt5*^−^/*Krt17*^+^ aberrant basaloid cells, *SPP1*^+^ macrophages, or *PVLAP*^+^ bronchial vessels are located, is lacking (*5*–*9*, *16*). The wide spectrum of IPF-associated cell types suggests the presence of multiple distinct niches in the IPF lung. Understanding the cellular and molecular interplay within each niche spatially is essential to better dissect unique disease pathobiology and guide next-generation drug discovery.

Spatial transcriptomics addresses this by providing spatially resolved gene expression within the natural tissue environment of intact tissue without inducing cell stress, death, or an isolation bias (*13*, *17*, *18*). The recently established Visium for formalin-fixed and paraffin-embedded (FFPE) technology enables an unbiased probe–based near whole-transcriptome wide mRNA analysis of preserved FFPE tissue sections, while the Xenium in situ technology allows a subcellular resolution for up to 500 selected genes (*14*, *19*). Previous analyses using RNA from small regions of interest had successfully compared IPF and control samples (*20*), but studies looking at the whole tissue slice are still missing.

In this study, we use these technologies to investigate the spatial transcriptomic profiles of IPF and control patient FFPE tissue sections. We also generated an integrated pulmonary fibrosis and interstitial lung disease (PF-ILD) scRNA-seq atlas from published datasets. Combining these two modalities, we used the scRNA-seq data to deconvolute the cell type composition of Visium spots to map well-defined cell types and genes onto the tissue. Through bioinformatic analysis, we found eight distinct cellular tissue microenvironments, or niches, with unique cellular composition and localization. Three of these were unique to IPF tissue: the fibrotic niche composed of myofibroblasts and aberrant basaloid cells, the airway macrophage niche containing osteopontin (SPP1)^+^ macrophages, and the immune niche containing mostly lymphoid cells, surrounded by deranged endothelial cells. We validated these niches using both multiplexed in situ mRNA imaging and high-plex sequential protein immunofluorescence. We then used the validated spatial cell type information to reconstruct the niche within the scRNA-seq data for a spatially educated and in-depth cell-cell communication analysis. This approach allowed us to reveal detailed tissue-based pathologic cellular cross-talk providing a resource for drug discovery aimed at disrupting disease-driving cellular niches in IPF.

## RESULTS

### Spatial and scRNA-seq profiling localizes cells and genes in healthy and IPF lung tissue

**Fig. 1.**
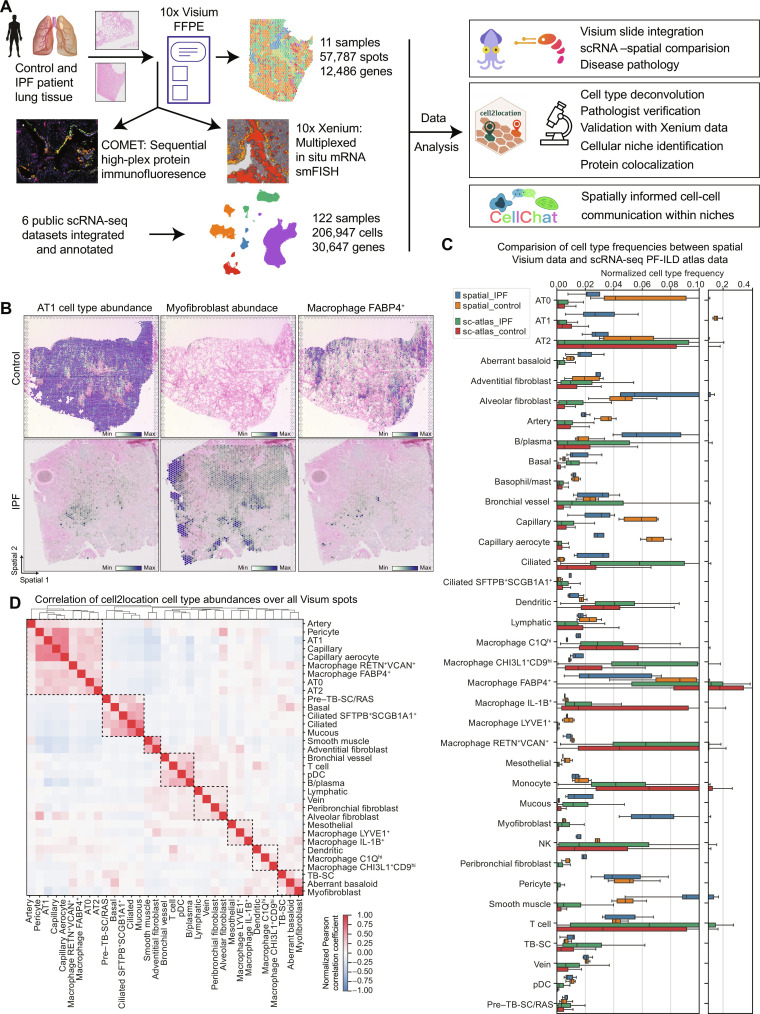
Combining spatial transcriptomics and scRNA-seq places genes and cell types to their tissue location. (**A**) Experimental design included spatial transcriptomics using Visium for FFPE and CytAssist (10x Genomics) on control and IPF patient samples (seven donors, 4× sections Visium CytAssist for FFPE, and 7× sections with Visium for FFPE; see fig. S1A for distribution of donors and sections). On two adjacent IPF and two control sections, as well as three additional IPF sections, we applied Xenium in situ (10x Genomics) for multiplexed in situ mRNA imaging similar to Single Molecule Fluorescence In Situ Hybridization (smFISH) and high-plex sequential protein immunofluorescence on the COMET platform. Six publicly available scRNA-seq datasets were integrated and annotated into a PF-ILD atlas. Visium and scRNA-seq data were combined for various analysis steps, including cell type deconvolution or cell-cell communication. (**B**) Spatial plots show cell2location mapping of estimated cell type abundance of AT1, *FABP4*^+^ alveolar macrophages, and myofibroblast cells on one control and one IPF tissue section. (**C**) Comparison of normalized cell type frequencies in spatial transcriptomic data and scRNA-seq PF-ILD atlas. (**D**) Pearson correlation of cell type abundances estimated with cell2location across all tissue sections identifies spatial colocated cell type modules.

To localize IPF disease-associated cell types in situ and gain insight into their cellular neighborhood, we performed spatial transcriptomics using the Visium for FFPE and CytAssist platforms (Fig. 1A). We profiled 11 hematoxylin and eosin (H&E)–stained tissue sections from three IPF and four control patients (fig. S1A and table S2). Spots from all slides were integrated into one data manifold, representing 57,787 spots with 12,486 consistently expressed genes, after sample processing and quality control (fig. S1B). We assessed the spatial distribution of cells in the tissue by estimating the cell type compositions of each spot, thereby increasing its resolution.

In parallel, we integrated six previously published scRNA-seq datasets (*4*–*6*, *8*, *9*, *21*) of ILDs and control tissue from human lungs into a PF-ILD atlas to ensure reproducibility and increase statistical power (as described in Materials and Methods) (Fig. 1A). For this study, focusing on IPF and nonfibrotic controls, our PF-ILD atlas represents gene expression profiles of 206,947 single cells from 122 human individuals (IPF, *n* = 65; controls, *n* = 57) across all six studies (fig. S2B). The final annotation, based on established single-cell signatures in the human lung (figs. S2, C to J, and S3, A to D) (*9*), represents 37 cell populations, characterized by distinct marker gene expression profiles, including recently reported pathologically preserved *SPP1*^+^ macrophages, *CTHRC1*^+^ myofibroblasts, *KRT5*^−^/*KRT17*^+^ aberrant basaloid cells, and *PVLAP*^+^ bronchial vessels (fig. S3, E and F, and table S1).

Using the cell2location package (*22*), each spot was deconvoluted on the basis of the annotated scRNA-seq data of the integrated PF-ILD atlas (Fig. 1A). Comparison of histopathological annotation by a pathologist on H&E-stained tissue sections and estimated cell type abundance confirmed successful mapping of well-described cell types to their expected location, such as arteries and veins or smooth muscle cells around large vessels, as well as ciliated and mucous cells in airways (fig. S1, C to E). Subtypes of fibroblast, sharing a similar transcriptome yet with few distinct marker genes, also mapped to their associated location, such as peribronchiolar fibroblasts around airways (fig. S4A). We observed disease-specific effects such as the decrease in AT1 cells and *FAPB4*^+^ alveolar macrophages and the emergence of *CTHRC1*^+^ myofibroblasts in IPF tissue (Fig. 1B).

To evaluate how well cell type frequencies were represented in spatial transcriptomics data, we compared the Visium data with the integrated PF-ILD scRNA-seq atlas (Fig. 1C). While mesenchymal cell type or airway epithelial cell type frequencies were comparable between the modalities, myeloid cells such as all macrophage subtypes were found with higher frequencies in scRNA-seq data. On the contrary, AT1 and aberrant basaloid cells or capillaries were better represented in the spatial data, validating the spatial transcriptomics approach for the analysis of healthy and diseased lung tissue.

We correlated the estimated cell type abundances per spot with each other to account for the resolution of multiple cells per spot (Fig. 1D). The clustering revealed colocalized cell types, representative of histopathological regions such as alveoli with AT1 cells, AT2 cells, capillaries, and pericytes, as well as airways with basal, ciliated, and mucous cells. In addition, the colocalized cell types indicated disease-induced changes with aberrant basaloid and myofibroblast clustering together or various immune cells such as T and B cells together with bronchial vessels.

To ultimately validate the deconvolution of spots into cell types, we applied Xenium multiplexed in situ hybridization to adjacent sections of two IPF and two control patient tissue blocks. We annotated the true single cells in the Xenium tissue using label transfer from the scRNA-seq PF-ILD atlas and grouped them into Visium spot–sized mRNA pseudo-bulks. Applying cell2location to deconvolute those pseudo-spots with the PF-ILD atlas as a reference revealed a high correlation between all the cell types and across tissue sections (fig. S4, B to D). In summary, the combination of nearly whole-transcriptome spatial transcriptomics together with an integrated scRNA-seq PF-ILD atlas enables the accurate localization and analysis of discrete cell types in their tissue context to understand the changes in IPF.

### Three distinct cellular niches appear only in IPF lung tissue

**Fig. 2.**
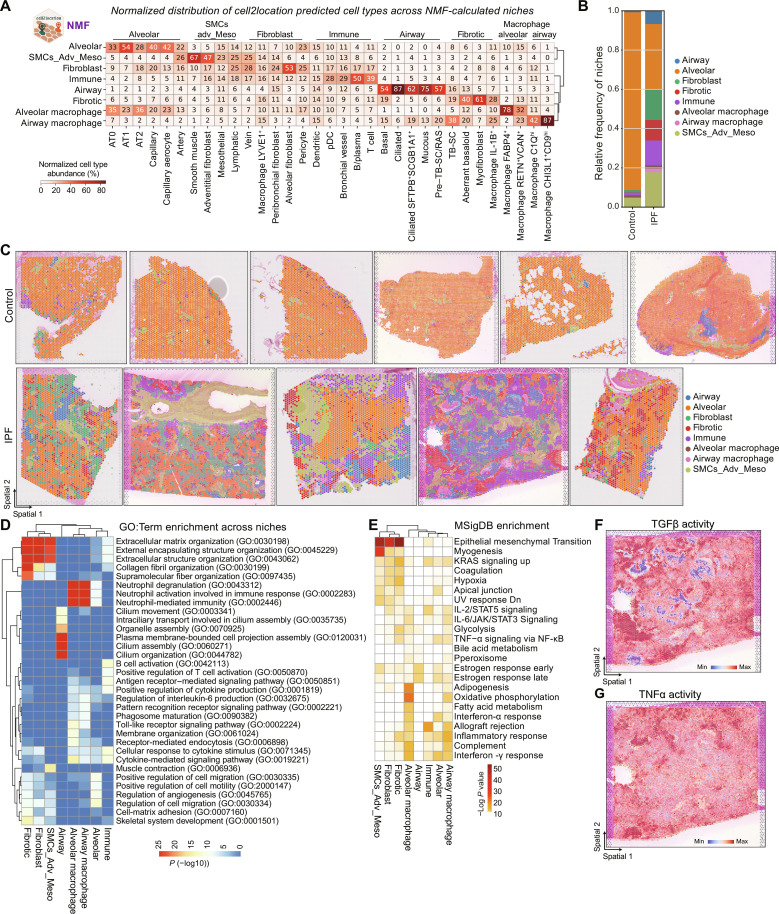
Identification of three characteristic cellular niches in IPF lung tissue. (**A**) Heatmap shows the normalized distribution of estimated cell type abundance in percentages across the niches, which were computed using the NMF method of the cell2location package. SMCs_Adv_Meso refers to smooth muscle, adventitial fibroblast, and mesothelial cells. (**B**) Relative frequency of niches across disease conditions is shown. Values are aggregated for four control and three IPF donors. (**C**) Spatial plots visualize the niche annotation across all samples and across conditions. (**D**) The heatmap shows top 3 enriched GO: terms per niche, colored by–log_10_ *P* value. (**E**) The heatmap shows top enriched Molecular Signatures Database (MSigDB) hallmark genes sets per niche. UV, ultraviolet; STAT5, signal transducers and activators of transcription 5; JAK, Janus kinase; NF-κB, nuclear factor κB; KRAS, Kirsten rat sarcoma virus; Dn, down-regulated. (**F** and **G**) Spatial plots exemplary show pathway activity for one IPF section.

To explore the spatial organization of lung tissue, we performed non-negative matrix factorization (NMF), a technique used to decompose multivariate datasets into a set of patterns and their corresponding weights. Here, we transform the cell2location spot–by–cell type output matrix into a matrix of cell types and niches, as well as spots and niches. This enables us to identify the predominant cellular niche, within each spot and the cell types that characterize each niche, abstracting the complexity of the biological system and identify the most relevant cellular neighborhoods within the tissue (*22*). Hypothesizing that cellular niches could serve as structural and functional building blocks composed of characteristic cell types, we used NMF to identify them in an unbiased manner across all sections and conditions. We then evaluated factors of co-occurring cell types (fig. S5A) and identified eight cellular niches with distinct cell type profiles and corresponding marker gene signatures (Fig. 2A, fig. S5B, and table S3).

We next assessed the relative frequency of these niches in control and IPF tissue. While control tissues were dominated by the alveolar niche, which mainly contains AT1 and AT2 cells and capillaries additional niches expected in the healthy lung were also observed in small quantities—these include the airway niche, the fibroblast niche, the smooth muscle/adventitial/mesothelial niche, as well as the alveolar macrophage niche (Fig. 2B). The array of healthy respiratory tissue niches could still be observed in diseased lungs—an indicator of the pathologic heterogeneity of pulmonary fibrosis (Fig. 2C).

The airway niche contained the highest portion of airway epithelial cells with basal, ciliated, *SFPTB*^+^*SCGB1A*^+^-ciliated cells, mucous, and pre–terminal bronchial secretory cells (pre–TB-SCs). The fibroblast niche contained the highest proportion of different fibroblast subsets, while the mixed smooth muscle/adventitial/mesothelial niche contained several structural cell types such as smooth muscle cells (SMCs), arteries, adventitial fibroblasts, or mesothelial cells. The alveolar macrophage niche, while containing the highest proportion of *FAPB4*^+^ alveolar macrophages and *RENT*^+^*/VCAN*^+^ macrophages, still contained decreased numbers of alveolar subtypes such as AT1 cells or capillaries but the highest proportion of AT2 cells (Fig. 2A).

In IPF, however, the alveolar niche frequency decreased markedly, with subsequent increases in all other niches observed (fig. S5C). Three niches were identified exclusively in IPF tissue, of which we assessed cell type frequencies both across and within the niches: (i) the fibrotic niche containing myofibroblasts (61% of total cell type, making up 34% within the niche), aberrant basaloid cells (40% of total, 9% in niche), and, unexpectedly, the airway-associated cell type known as TB-SCs (19% of total, 3% in niche) (*23*); (ii) the immune niche containing B cells (50% of total, 24% in niche) and T cells (39% of total, 14% in niche), dendritic cells (15% of total, 2% in niche), and *PVLAP*^+^ ectopic endothelial cells—here called bronchial vessels (29% of total, 6% in niche); and (iii) the airway macrophage niche containing *SPP1*^+^ macrophage subsets of *CHI3L1*^+^*CD9*^hi^ (87% of total, 57% in niche) and *C1Q*^hi^ macrophages (42% of total, 5% in niche), as well as TB-SCs (38% of total, 4% in niche) (Fig. 1, B and C; and figs. S5, C to E, and S6).

To link cellular function to spatial cell composition, we performed enrichment analysis on spatial niches. We used differential gene expression analysis to identify niche-specific gene signatures between niches and did functional analysis using Molecular Signatures Database (MSigDB) hallmark gene sets and gene ontology (GO) and estimated signaling pathway activities (PROGENy and decoupleR) (*24*) for all spots in one niche.

For example, the fibrotic niche can be characterized with several extracellular matrix (ECM)–associated GO terms and MSigDB terms such as epithelial mesenchymal transition or hypoxia and colocalized with areas of highest transforming growth factor–β (TGFβ) signaling activity and myofibroblast occurrence (Fig. 2, D to F, and fig. S5F). Airway macrophage niches were the areas of high tumor necrosis factor–α (TNFα) signaling activity that colocalized with *SPP1*^+^ macrophages and could be characterized with terms referring to neutrophil activation, interferon-γ response or interleukin-6 (IL-6) regulation (Fig. 2, D and F, and fig. S5, D and E).

To explore the spatial organization of identified niches, we leveraged the positional data and analyzed neighboring interactions. While the control tissue was dominated by the alveolar niche, in IPF, the tissue appeared disorganized, both when looking at the immediate (1-ring) or extended neighborhood (3-rings) of a spot (fig. S5, G and H). The fibrotic niche seemed to neighbor all other niches, especially alveolar, immune, fibroblast, and airway niches (fig. S5I). In summary, the unbiased identification of cellular niches as functional tissue building blocks allowed the analysis of spatial organization across samples and the changes in disease, which revealed the presence of characteristic IPF tissue specific fibrotic, immune, and macrophage niches.

### Aberrant basaloid cells are found around airways

**Fig. 3.**
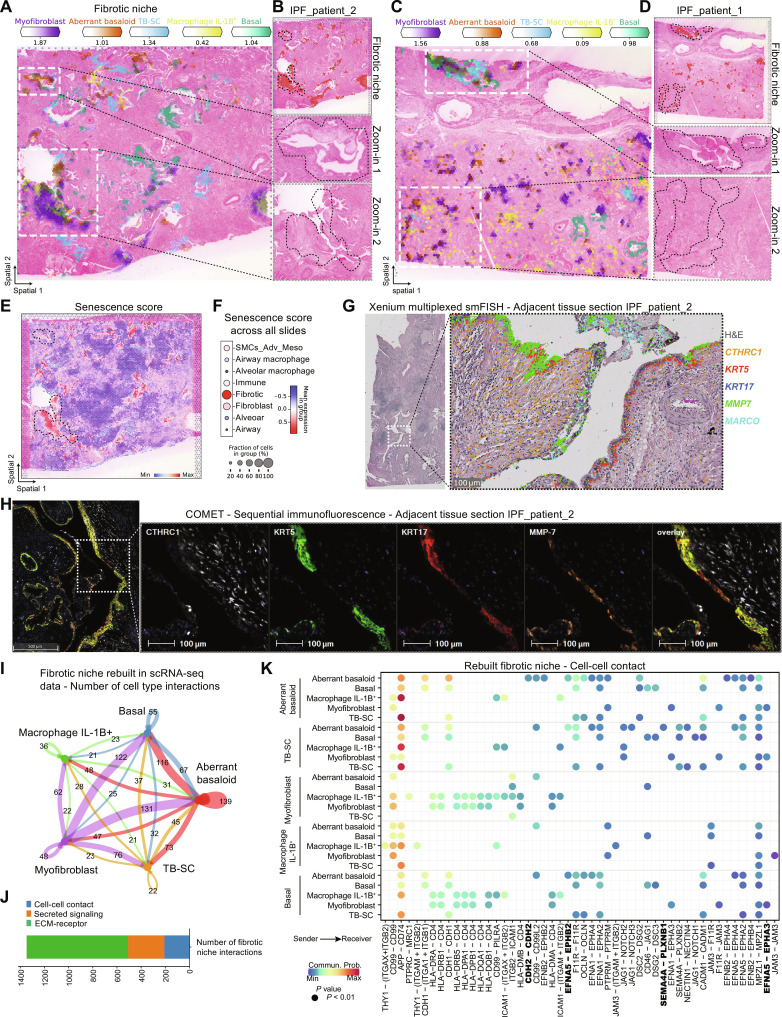
The fibrotic niche localizes around airways. (**A** to **D**) Spatial plots show, for two IPF tissue sections, (A and C) the abundance of fibrotic niche–associated cell types and (B and D) the fibrotic niche distribution and zoom into H&E-stained tissue for highlighted regions. (**E** and **F**) Senescence gene score (*CDKN1A, CDKN1B, CDKN2B, TP53, SERPINE1,* and *GLB1*) calculated using hotspot gene-module scoring (*29*) (E) on one IPF tissue section and (F) as dot plot against the other niches. (**G**) Xenium mRNA in situ hybridization data on an adjacent tissue section from (A). (**H**) Multiplexed protein immunofluorescence on an adjacent tissue section from (A). (**I**) Bubble plot summarizing the number of interactions within the rebuilt fibrotic niche in the scRNA-seq PF-ILD atlas data. (**J**) Number of interactions within the fibrotic niche per communication category. (**K**) Heatmap of statistically significant ligand-receptor pairs from the cell-cell contact category within the fibrotic niche.

To further study the IPF-specific fibrotic niche, we first looked at the distribution across patients, confirming prevalence in all IPF tissue sections (fig. S6A). Next, we focused on the cell types contained within the niche based on the NMF and the frequency analysis to also account for cell types making up a small portion of a niche but contributing with a large percentage of its total amount (Fig. 2A and fig. S5, D and E). We verified colocalization of myofibroblast, aberrant basaloid cells, TB-SC, basal cells, and *IL1B*^+^ macrophages, shown by way of example on two IPF tissue sections (Fig. 3, A to D; and figs. S10, A to C, and S11, A and C). Overlap of established cell type marker genes such as *CTHRC1* and *ASPN* for myofibroblasts, *MMP7* and *KRT17* for aberrant basaloid cells, as well as secretoglobin family 3A member 2 (*SCGB3A2*) *SCGB3A2* for TB-SC additionally validated these data (fig. S11, B, D, and E). Unexpectedly, we found the fibrotic niche preferentially located around airways. Myofibroblasts could also be found alone in more alveolar regions, which agrees with previous reports that found IPF-specific fibroblastic foci predominantly in the more damage-susceptible distal parenchyma (*23*, *25*). Aberrant basaloid cells visually localized to airways and were always found together with myofibroblasts (Fig. 3, B and D, and fig. S10, A and B). Supporting this finding, aberrant basaloid cell abundance correlated with a fraction of basal cells and with recently identified TB-SC cells that reside in the distinct location of terminal bronchi, an anatomical feature found in human but not in mice (Figs. 1D and 2A and fig. S5A) (*26*). Aberrant basaloid cells have previously been associated with senescence pathways (*8*, *9*, *27*, *28*). We therefore calculated a custom senescence gene score for every spot in the spatial data using the hotspot gene-module scoring (*29*), visualized as dot plot across the niches, and on one tissue section (*30*, *31*). The score was the highest in the fibrotic niche and in the highlighted regions of myofibroblast and aberrant basaloid colocalization (Fig. 3, E and F, and fig. S11, E and F).

Accounting for the fact that Visium spots capture multiple cells, we set out to validate our findings with true single-cell technologies. First, we applied Xenium multiplexed mRNA in situ hybridization to validate cell type marker gene colocalization in the same area on adjacent tissue section of patient_IPF_1 and patient_IPF_2 (Fig. 3G and fig. S7, B and F), as well as on sections of three additional patients with IPF (fig. S8, B, F, and J). We demonstrated colocalization of *KRT5*^−^*KRT17*^+^ aberrant basaloid cells with *CTHRC1*^+^ myofibroblasts next to *KRT5*^+^*KRT17*^+^ basal cells, *MMP7*^+^ airway cells, and macrophage receptor with collagenous structure (*MARCO*)–expressing macrophages (Fig. 3G). To make the link toward functional proteins, we applied COMET high-plex sequential immunofluorescence onto adjacent tissue sections (fig. S9, A and C). Again, we observed the colocalization of KRT5^−^KRT17^+^ aberrant basaloid cells with CTHRC1^+^ myofibroblast next to KRT5^+^KRT17^+^ basal cells and matrix metalloproteinase 7 (MMP7)^+^ airway cells (Fig. 3G). Thus, we validated the fibrotic niche and the cell types therein with three technologies and on both mRNA and protein levels.

To further understand the fibrotic niche and the mechanism that regulated the dynamics of this cellular niche, we used CellChat (*32*) to perform cell-cell communication analysis. Making use of knowledge and confidence gained from the three spatial modalities, we used the spatially validated cell types to rebuild the fibrotic niche in the higher-resolution scRNA-seq data of the PF-ILD atlas (table S4). Summarizing the number of all interactions revealed that aberrant basaloid cells would mostly interact with myofibroblasts, directly followed by basal cells and TB-SCs, providing further evidence for their airway such as character (Fig. 3I).

Focusing on the cell-cell contact interactions between validated colocated cell types, we identified the ephrin pathway as major contributor, besides cadherin (CDH) and notch signaling (Fig. 3K and fig. S12, A and E). The ephrin pathway has previously been associated with proliferative diseases such as cancer, yet we found distinct interactions within the fibrotic niche, suggesting a potential role in fibrosis (*33*). The receptor *EPHB2* was specifically expressed by aberrant basaloid cells, whereas the receptor *EPHA3* seemed to be specific for myofibroblasts. Both received signals from *EFNA5*, mostly expressed by airway epithelial cell populations (Fig. 3K and fig. S12, B and C). Both observations could be validated in the spatial data, with *EPHA3* and *EPHB2* mostly expressed in the fibrotic niche, while the ligand *EFNA5* is up-regulated in IPF and mostly expressed in the airway niche (fig. S12D).

In terms of secreted signaling, midkine signaling was found to be the most prevalent in the fibrotic niche, followed by known fibrosis pathways such as TGFβ, epithelial growth factor, semaphorin, or bone morphogenic protein (BMP) (fig. S12, A and E) (*34*). The potential for BMP signaling to influence on aberrant basaloid cells became apparent by the interaction between peribronchial and alveolar fibroblasts, as well as myofibroblasts, with the ligand *BMP5* and the dimerizing receptors *ACVR1* and *BMPR2*, only expressed by aberrant basaloid cells (fig. S8C).

Cell-cell communication within the fibrotic niche could be attributed to ECM-receptor–associated ligand-receptor pairs—over 60% of which could be assigned to collagen, laminin, thrombospondin, and fibronectin pathways (Fig. 3J and fig. S12A). While myofibroblasts were responsible for most of these ECM interactions, aberrant basaloid cells also contributed with the expression of fibronectin (*FN1)* or tenascin (*TNC*), consolidating their characteristic epithelial-to-mesenchymal transitional (EMT) features unique among the epithelial cells (fig. S13, A to C). However, they were also able to receive ECM signals, for example, from *TNC* via the expression of the integrin receptors *ITGAV* and *ITGB6* (fig. S13D) (*8*, *9*). Overall, the combination of unbiased transcriptomics and tissue structure revealed a preferential localization of the fibrotic niche and especially aberrant basaloid cells around airways and validated at the mRNA and protein level to allow a spatially educated cell-cell communication interactome, which describes pathology-driving signaling in these fibrotic areas.

### SPP1^+^ macrophage subsets localize to lumen of fibrotic airways

To further study the airway macrophage niche, we first aimed to understand the heterogeneity of cellular macrophage subtypes in IPF that all shared expression of *SPP1*—recognized as a common fibrotic macrophage marker across many fibrotic diseases (*5*, *35*). In our annotations, we refer to SPP1^+^ macrophages as *CHI3L1*^+^ macrophages, resembling previously identified *TREM2*^+^ macrophages (*36*), define the airway macrophage niche (Fig. 2A), which, together with *C1Q*^hi^ macrophages (*36*), was nearly exclusively found in IPF tissue (fig. S14, A and B). C1Q macrophages, while contributing 42% to the airway macrophage niche, were also found throughout the tissue including fibroblast and immune niches (Fig. 2A and fig. S14B). IL-1B^+^ macrophages colocalized with both the fibrotic niche and the airway macrophage niche in distinct histopathological regions (fig. S14C).

**Fig. 4.**
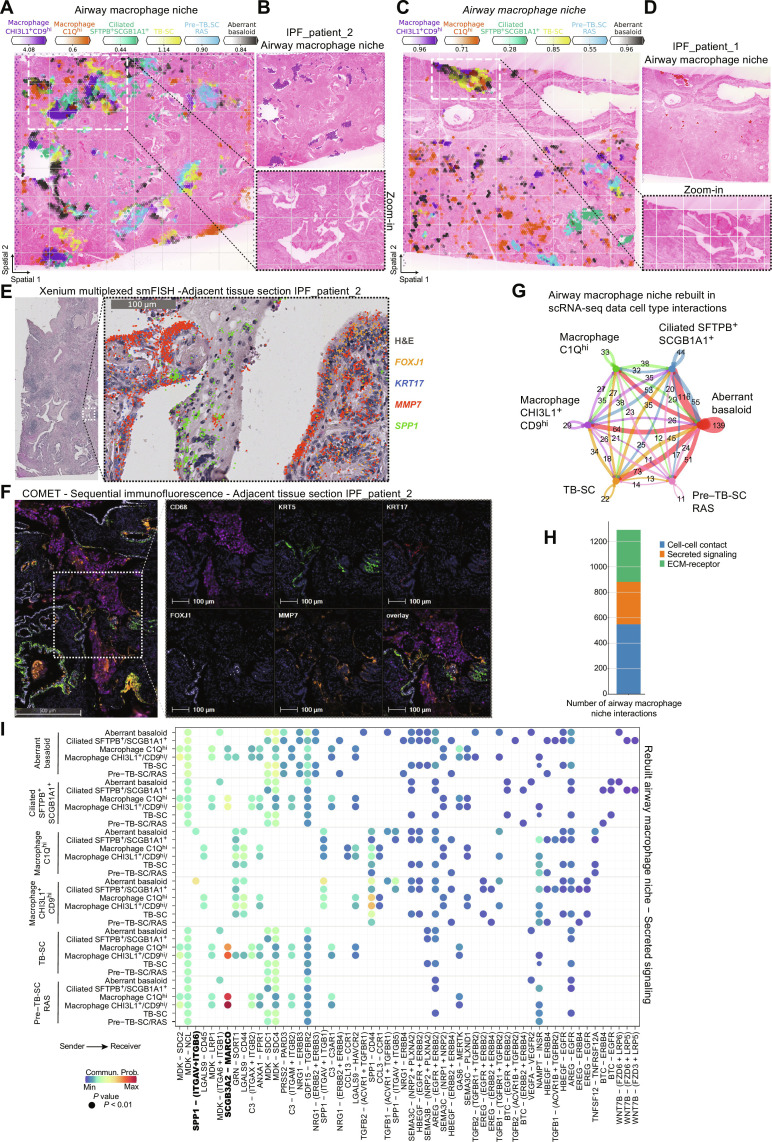
SPP1^+^ macrophages are localized to fibrotic airway lumen. (**A** to **D**) Spatial plots show, for two IPF tissue sections, (A and C) the abundance of airway macrophage niche associated cell types and (B and D) the niche distribution and zoom into H&E-stained tissue for highlighted regions. (**E**) Xenium mRNA in situ hybridization data on an adjacent tissue section from (A). (**F**) Multiplexed protein immunofluorescence on an adjacent tissue section from (A). (**G**) Bubble plot summarizing the number of interactions within the rebuilt airway macrophage niche in the scRNA-seq PF-ILD atlas data. (**H**) Number of interactions within the airway macrophage niche per communication category. (**I**) Heatmap of statistically significant ligand-receptor pairs from the secreted signaling category within the airway macrophage niche.

Next, we focused on the cell types contained in the niche based on the NMF analysis (Fig. 2A) and verified colocalization of *CHI3L1*^+^ and *C1Q*^hi^ macrophages together with aberrant basaloid cells, TB-SC, and *SFTPB*^+^-ciliated cells in IPF tissue sections (Fig. 4, A to D, and fig. S15, A and C). Overlap of established cell type marker genes *CHI3L1* and *CHIT1*, or *C1QA* and *CCL13* for macrophages, as well as *CAPS*, *SFTPB*, and *SCGB1A1* for ciliated cells, provided additional validation (fig. S15, B, D, and E).

We revealed that the airway macrophage niche was preferentially located inside the lumen of distal airways. *SFTPB* had recently been identified to discriminate distal airway epithelium from proximal cell populations (*26*), supporting our finding that the airway macrophage niche colocalizes with *SFTPB*^+^-ciliated cells but not *SFTPB-*ciliated cells, in what appear to be rather large yet distal airways (Figs. 2A and 4A and fig. S15). Having identified a similar location for the fibrotic niche, we looked at the distribution of both niches across the IPF sections. The airway macrophage niche, located inside the lumen, neighbored the fibrotic niche in parenchymal tissue areas adjoining the airway (fig. S15F). Checking the cell type distribution across the niches, myofibroblasts were rarely found outside the fibrotic niche, but 20% of the aberrant basaloid cells also contributed to the airway macrophage niche, verified by colocalization (Figs. 2A and 4, A and C).

We validated and extended our characterization of the airway macrophage niche, using both Xenium multiplexed mRNA in situ hybridization and multiplexed COMET protein immunofluorescence on adjacent tissue sections (figs. S7, C and G, and S9, B and D), as well as on sections of three additional patients with IPF (fig. S8, C, G, and K). On the mRNA level, we demonstrated colocalization of cell type marker genes with *SPP1*^+^ macrophages inside the airway lumen, lined with *FOXJ1*^+^-ciliated cells, *KRT17*^+^ TB-SCs, and *MMP7*^+^ airway cells (Fig. 4E). On the protein level on adjacent tissue sections (fig. S9, B and D), we observed an airway lined with FOXJ1 for ciliated cells, KRT17 for TB-SC, and MMP7 for airway cells and the airway lumen filled with CD68^+^ macrophages (Fig. 4F).

To further analyze the airway macrophage niche, we rebuilt it again with its contributing cell types in the scRNA-seq dataset and performed cell communication analysis (table S5). Summarizing the number of all interactions revealed that aberrant basaloid cells with their mixed epithelial and mesenchymal characteristics were showing the most interactions within the niche, connecting with *SFTPB*^+^-ciliated cells, TB-SC and *CHI3L1*^+^ macrophages (Fig. 4G). Cell-cell communication in the airway macrophage niche could mostly be assigned to cell-cell contact interactions (44%), followed by ECM-receptor (fig. S16A) and secreted signaling interactions (fig. S17B), in contrast to the more ECM-dominant interactions within the fibrotic niche (Fig. 4H). Most of the cell-cell contact–associated ligand-receptor pairs could be assigned to the major histocompatibility complex II pathway (fig. S16A). As an example, the interaction of *HLA-DMB* expressed by all the macrophage subtypes was shown to interact with *CD4*, expressed on ciliated cells and myofibroblasts, supporting the airway localization of the airway macrophage niche and its potential pathologic role (fig. S16, F and G).

The interactions in the secreted signaling also offer possible explanations for the airway localization. *SCGB3A2* expressed by the airway epithelial cell types within the niche, including aberrant basaloid cells, binds to the *MARCO*, expressed by all macrophages (fig. S16, B and E). In addition, the ligand *SPP1* can bind to dimerizing pairs of integrins *ITGAV* and *ITGB6*, expressed by aberrant basaloid cells, suggesting a distinct communication between these two cell types only arising under fibrotic conditions (fig. S16C). Aberrant basaloid cells seemed further stimulated by specific WNT signaling, secreted by mucosal and SFTPB^+^-ciliated cells in the form of WNT7B, binding to the dimerizing receptor of *FZD6* and *LRP6* (fig. S16D). All three ligand-receptor observations could be validated in the spatial data, with *SCGB3A2* and *SPP1* mostly expressed in the airway macrophage niche in IPF, while *MARCO* and the integrins *ITGAV* and *ITGB6*, as well as the WNT receptors *FZD6* and *LRP6*, were up-regulated in IPF and expressed in the airway macrophage and airway niche, respectively (fig. S16G).

Overall, our spatial Visium data revealed the differential localization of macrophage subtypes in IPF and suggested a preferential localization of the airway macrophage niche to the lumen of fibrotic airways, which we could validate on mRNA and protein level. The airway macrophage niche also appeared in proximity to the fibrotic niche and especially aberrant basaloid cells—supported by predicted and distinct cell-cell communication.

### Immune cell foci are recruited by IPF-specific ectopic endothelial cells

**Fig. 5.**
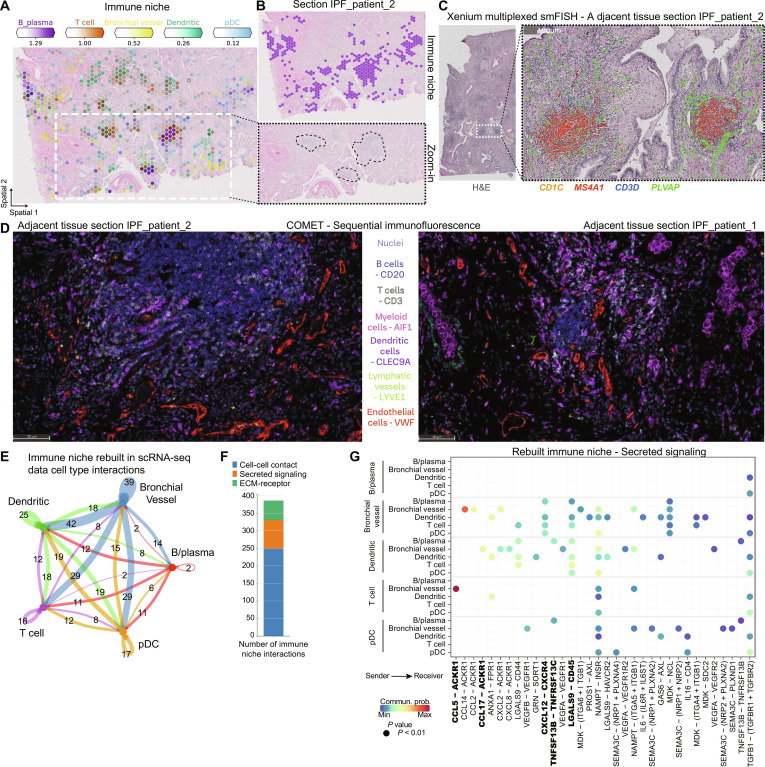
Immune cells from foci are recruited by IPF-specific bronchial vessels. (**A** and **B**) Spatial plots show, for one IPF tissue section, (A) the abundance of immune niche–associated cell types and (B) the immune niche distribution and zoom into H&E-stained tissue for highlighted regions. (**C**) Xenium mRNA in situ hybridization data on an adjacent tissue section from (A). (**D**) Multiplexed protein immunofluorescence on an adjacent tissue section from (A). (**E**) Bubble plot summarizing the number of interactions between cell types within the rebuilt immune niche in the scRNA-seq PF-ILD atlas data. (**F**) Number of interactions within the immune niche per communication category. (**G**) Heatmap of statistically significant ligand-receptor pairs from the cell-cell contact category within the immune niche.

The third pathologic niche appearing in all IPF tissue sections was the immune niche, consisting of B and plasma cells, T cells, plasmacytoid dendritic cells (pDCs), and the recently found *PLVAP*^+^ ectopic endothelial cell population—here named bronchial vessels (fig. S5B) (*16*, *37*, *38*). While all cell types contributing to the immune niche show a marked increase in IPF tissue compared to healthy controls (fig. S18), we noticed them clustering together in distinct foci, even visible in the H&E staining by a gray color (Fig. 5, A and B). We verified colocalization of the contributing cell types in IPF tissue sections (Fig. 5, A and B, and fig. S19, A and C), as well as overlap of established cell type marker genes such as *CD19*, *CD79A*, and *MS4A1* for B and plasma cells, *CD3D* for T cells, and *PLVAP* for bronchial vessels (fig. S19B).

We further validated and extended our findings within the immune niche, using both Xenium multiplexed mRNA in situ hybridization and multiplexed COMET protein immunofluorescence on adjacent tissue sections (fig. S7, D and H), and on sections of three additional patients with IPF (fig. S8, D, H, and L). On the mRNA level, we demonstrated colocalization of cell type marker genes with *MS4A1* for B and plasma cells, *CD3D* for T cells, and *CD1C* for dendritic cells within distinct foci, surrounded by *PLVAP* for ectopic endothelial bronchial vessels (Fig. 5C).

On the protein level on adjacent sections of IPF_patient_1 and IPF_patient_2, we observed similar foci, consisting of CD20^+^ B cells, CD3^+^ T cells, and CLEC9A^+^ dendritic cells, lined by Von Willebrand factor (VWF)^+^ endothelium. We did not observe LYVE1^+^ lymphatic endothelial cells in the area (Fig. 5D).

We considered how the cells could organize in these foci and analyzed the cell-cell communication by rebuilding the niche again with its contributing cell types in the scRNA-seq dataset (table S6). Summarizing the number of all interactions revealed that the bronchial vessels appeared pivotal, contributing the most to the ligand-receptor interactions within the niche and with all the other cell types (Fig. 5E). The cell-cell communication in the immune niche mostly came from cell-cell contact interactions (66%) (fig. S20A), followed by secreted signaling (Fig. 5, F and G) and ECM-receptor interactions (fig. S20B).

Focusing on secreted signaling, we identified bronchial vessels as secreting several chemokines and cytokines able to recruit lymphoid cells (Fig. 5G). As an example, bronchial vessels expressed *CXCL12* also found on myofibroblasts, which bind to the receptor *CXCR4*, and found on T cells, B cells, pDCs, and dendritic cells (fig. S19E). However, bronchial vessels, with their receptor *ACKR1*, seem also to be able to act upon signals via *CCL17* from dendritic cells or *CCL5* from T cells and natural killer (NK) cells (fig. S19, F and G). These observations could be validated in the spatial data, with *ACKR1* and *CXCR4* being most abundant, which, together with *CXCL12*, *CCL17*, and *CCL5*, were highly expressed in the immune niche (fig. S19H). Overall, the spatial transcriptomics data, together with H&E tissue structure, revealed the immune niche as a notable histopathological feature in IPF, in the form of foci of multiple lymphoid cell types, potentially recruited by distinct secreted signaling of the ectopic endothelial bronchial vessels.

## DISCUSSION

We integrated six independently published human lung scRNA-seq studies into a harmonized PF-ILD atlas using state of the art computational tools. This provided us with a robust and generalizable resource of patients with IPF at a single-cell level. Simultaneously, we performed spatial transcriptomic analysis of lung FFPE tissue, comparing spatially resolved gene expression between patients with IPF and control patients. In this study, we mainly made use of the high-confidence cell type annotation derived from scRNA-seq data that enabled an increased resolution of the spatial transcriptomics data, by estimating the cell type abundance per spot. Comparing cell type frequencies between the modalities, confirmed a cell type bias in tissue dissociation and sample processing for scRNA-seq: Fragile epithelial and endothelial cell types, as well as ECM-embedded myofibroblasts, were more abundant in the spatial Visium data, while robust myeloid cells such as macrophages were overrepresented in scRNA-seq data. This bias is also observed when comparing single-nucleus data with scRNA-seq data, which also does not require cell isolation, and validates spatial transcriptomics as a tool to capture biology in situ (*14*, *15*, *39*).

We used NMF to identify three cellular niches that only exist in fibrotic IPF tissue: the fibrotic niche, the airway macrophage niche, and the immune niche. Our analysis revealed a disorganized tissue structure compared to control sections. However, we identified a preferential localization of the fibrotic niche and especially the aberrant basaloid cells around airways in the IPF tissue. Aberrant basaloid cells, as a more recently identified disease-associated cell state of unknown and debated origin, were first characterized as distinct from any epithelial cell type due to their mesenchymal and EMT marker expression and associated senescence profile (*8*, *9*). Here, we demonstrate a colocalization of aberrant basaloid cells with myofibroblasts around airways that correlated with a senescence gene-signature score.

To validate the identified niches, we used two different state-of-the-art methodologies: multiplex RNA in situ hybridization using the Xenium platform and high-plex sequential protein immunofluorescence using the COMET platform. Both technologies offer single-cell resolution in the tissue context, albeit for a limited number of genes or proteins. We were able to confirm colocalization of cell type marker genes and proteins for all three pathological niches, as identified from the analysis of the Visium data.

Subsequently, we turned to cell-cell communication analysis. Traditionally, this has been used primarily as a discovery tool to unveil potentially interacting cell types based on ligand-receptor pair expression, often without considering whether those cell types are close enough to interact. Our niche validations confirmed that cell types within a niche are close enough to interact. This insight enabled us to conduct a spatially informed cell-cell communication analysis using our single-cell IPF atlas, focusing only on the colocalized cell types found in the identified pathological niches.

In the fibrotic niche, aberrant basaloid cells and myofibroblasts were found to be heavily involved in the ephrin pathway based on receptor-tyrosine kinase signaling, so far only associated with proliferative disease such as cancer (*33*). While proliferation in the fibrotic niche could be characteristic of fibroblasts, this is in contrast to the senescent nature of aberrant basaloid cells, calling for further research of the ephrin pathway and the interacting cells within the fibrotic niche (*27*, *40*, *41*).

A disease-relevant finding from this study is the identification of a specific type of macrophages, located in the lumen of airways that are exclusive to fibrotic tissue. Previous single-cell analyses identified a unique IPF macrophage subpopulation characterized by SPP1 expression (*5*) [here referred as *CHI3L1*^+^ macrophages and comparable to previously identified *TREM2*^+^ macrophages (*36*)]. In this study, notably, we located these macrophages in the airways of IPF tissue, where they colocalize with distal ciliated cells, TB-SCs in the airway lumen, and aberrant basaloid cells. A review of existing literature revealed that airway macrophages have been reported in mouse models (*42*), although the markers identified in these models do not match those found in humans, indicating a need for further research in this area. Our cell-cell communication analysis sheds some on the relatively unexplored area of interactions between macrophages and airway epithelium in disease (*43*). The origin of these fibrotic airway–associated macrophages and whether varying functions are associated with different localizations requires further exploration (*5*). Airway macrophage and fibrotic niches both occupy a similar neighborhood in and around the large, but distal, airways of fibrotic lungs, which appear distinctly different from remodeled small airways in highly fibrotic parenchymal tissue (*7*). The fact that aberrant basaloid cells seem to be split between these niches, in both cases localized to and interacting with the distal airway epithelium, suggests a potential airway origin of this intriguing, disease-associated cell type (*44*).

We identified the immune niche as histologically visible foci of immune infiltrates, predominantly consisting of adaptive immune cells, especially B/plasma and T cells (*45*). These immune cells colocalized with the recently identified ectopic *PLVAP*^+^ (also *COL15A1*^+^) endothelial cells often referred as bronchial vessels (*16*). While previous studies report an association of these endothelial cells to major airways (*16*), we find no preferential localization of the immune niche or bronchial vessels in the tissue. Our analysis confirmed an increased abundance of bronchial vessels in IPF, which were better represented in the spatial data than in the scRNA-seq data (Fig. 1C). Of note is their potential ability to recruit the immune cells into those foci, via the *CXCL12*-*CXCR4* axis according to the communication analyses, thereby indicating an important role of the endothelium into the aberrant remodeling of the distal parenchymal tissue in IPF (*46*). This is in line with our previously reported findings on the fibrovascular interface in other fibrotic pulmonary diseases (*47*, *48*).

Our initial analysis, based on Visium technology, revealed the following limitations, which we addressed during the validation of the results: the small number of samples, the resolution of the Visium spot size (50 μm), and the reliance on RNA data only. The Visium analyses were conducted on tissue from four controls and three IPF samples. By incorporating three additional IPF donors for Xenium samples, we did not identify any new niches, but we were able validate the Visium results. Furthermore, our cell-cell communication results were based on 65 IPF samples and 57 controls. Therefore, despite the small sample size, we argue that the reported results serve as a foundation for more extensive studies in the future when the currently high costs and associated complexity of the technologies decrease. These studies will benefit from using a wider group of patients at different disease stages, beyond end-stage IPF, and across a broader selection of tissue locations in the lung. Regarding the Visium resolution, we demonstrated that algorithms to deconvolute data derived from 50-μm Visium spots into cell type proportions are powerful tools when a well-annotated single-cell data reference is available (*22*, *49*–*51*). Our validations show a strong correlation between predicted cell type fractions and a gold standard generated using pseudo-Visium–sized spots, compared with the actual cell type fractions in a Xenium in situ dataset with subcellular transcript resolution. Last, to assess the RNA results, which can differ from their functional role defined by protein expression, we used high-plex immunofluorescence to validate distinct cell type markers at the protein level. While this approach can only work on a handful of proteins, further technological advancements are needed to cover a larger fraction of the proteome in a spatial manner. The upcoming wave of subcellular resolution techniques (*51*, *52*) can move the field even closer to an in situ single-cell analysis in the natural tissue context.

Despite these limitations, our data provide key starting points for drug discovery in IPF, where a profound unmet medical need persists beyond the standard antifibrotic treatments, such as nintedanib (*53*). Traditional tactics to find new targets rely on observing changes in gene expression between healthy and diseased tissue (often bulk RNA-seq), followed by in vitro validation experiments using a single-cell type responding to a target-relevant stimulus. New therapies are needed that recognize the importance of different disease-associated cell populations beyond isolated myofibroblasts and address the cell-cell miscommunication between those populations, which drive pathology (*54*). In this study, we spatially characterize three key disease-specific niches and reveal the lines of communication within these niches. This data can guide the design of in vitro models that accurately recapitulate the disease niche context, thereby facilitating the development of more precise test cascades to validate targets aiming at disrupting disease-driving niches.

## MATERIALS AND METHODS

### Integration of public scRNA-seq datasets into PF-ILD atlas

Single-cell count matrices containing interstitial lung diseases and control samples were identified and downloaded from Gene Expression Omnibus (GEO). Samples were processed following best practices (*55*) using SCANPY (*56*) and integrated using scVI (*57*). For integration of data, we used the union of all common genes between the datasets and then selected 6500 highly variable genes using the Seurat v3 method. The individual single-cell samples were used for batch correction and the percentage of mitochondrial genes as continuous covariate and the sample publication as categorical covariate. While mitochondrial percentage is a common covariate used, we reasoned that batch effects from each sample and from the laboratory differences in tissue dissociation and processing steps in the laboratory and in silico should be considered. The parameters used were as follow: n_hidden: 128, n_latent: 10, n_layers: 1, and dropout_rate: 0.1. The integrated dataset contains 405,741 cells from 155 samples for the following diagnosis: IPF (*n* = 65), chronic obstructive pulmonary disease (COPD; *n* = 17), systemic sclerosis-associated interstitial lung disease (SSc-ILD; *n* = 12), myositis-ILD (*n* = 1), hypersensitivity pneumonia (*n* = 1), COVID (*n* = 3), and control (*n* = 57). For analysis in this manuscript, an IPF version of the atlas was used, subsetting the PF-ILD atlas by diagnosis to only IPF and control patients and downsampling the number of cells to maximum of 1000 per sample to reduce representation bias from studies with more cells.

### Cell type annotation

Cell lineages on the dataset were identified using broad clustering in combination with well-known markers as follows: epithelial (*EPCAM*), endothelial (*CLDN5*), mesenchyme (*COL1A1*), and immune (CD45—official gene name: *PTPRC*) that we subdivide into myeloid (*FCER1G*) and lymphoid (*FCER1G*-negative). For further annotations, the cell lineages were analyzed individually on the basis of well-known markers and the markers from the original dataset publications (for markers used to annotate cell types, see table S7) (*5*, *8*, *9*, *58*–*60*).

### Human tissue and ethics statement

Human FFPE tissue was sourced from a commercial vendor (BioIVT). Fresh human lung tissue from explants and resections was collected at Hannover Medical School. All organs were picked up at the operation theater immediately after surgery and worked up using standardized protocols as described elsewhere (*61*). Briefly summarized, obtained samples were fixed in 3.7% formaldehyde, subsequently dehydrated, and eventually embedded in paraffin by means of an automatic tissue-processing device (LOGOS microwave hybrid tissue processor, Milestone Medical, Sorisole, Italy). All patients gave written informed consent. The study was approved by and conducted according to the requirements of the local ethics committee at Hannover Medical School (8867_BO_K_2020).

### Tissue preparation for spatial transcriptomics

Tissue sections were prepared according to the Visium Spatial Gene Expression for FFPE (CG000408) or Visium CytAssist Tissue preparation guide (CG000518). Briefly, after cooling and rehydration of tissue blocks in an ice bath, 5-μm sections were prepared and transferred to a ribonuclease-free water bath at 42°C. Sections were placed on either Visium Spatial Gene Expression Slides directly (10x Genomics, standard workflow) or SuperFrost Plus microscopy slides (CytAssist workflow) and dried for 3 hours in an oven at 42°C, followed by overnight drying at room temperature.

Deparaffinization and H&E staining were performed according to Visium user guides (CG000409 and CG000520). Sections were incubated for 2 hours at 60°C in a dry oven before deparaffinization in xylene, and rehydration using an ethanol gradient was performed. Sections were mounted using 85% glycerol for the H&E imaging step.

H&E-stained slides were scanned on a Leica Axioscan bright-field scanner using ×20 magnification. Coverslips were removed by immersing the mounted slides in Milli-Q water until the coverslip detaches.

Using the freehand annotation function in HALO Link (Indica Labs), examples of distinct histological structures (arteries, veins, airways, fibroblastic foci, and muscle) were annotated by a pathologist.

### Visium spatial transcriptomics and sequencing

Following imaging, 11 H&E-stained tissue sections from three patients with IPF and four control patients were processed with the standard Visium spatial for FFPE gene expression kit, human transcriptome (seven sections, 10x Genomics, catalog no. 1000334) or the Visium CytAssist Spatial Gene Expression for FFPE, human transcriptome, 11 mm (four sections, 10x Genomics, catalog no. 1000444) as per the manufacturer’s instructions (CG0000407, Rev D; CytAssist CG000495, Rev. C). Figure S1A shows the tissue sections and donor distribution. Briefly, deparaffinized and de–cross-linked H&E-stained sections were hybridized with human wild-type probes (10x Genomics) directly on Visium slides (standard) or on SuperFrost Plus microscopy slides (CytAssist) on a thermal cycler at 50°C for ~18 hours, followed by probe ligation, CytAssist-enabled RNA degradation and tissue removal (CytAssist workflow only) extension, elution, and library preparation as per the manufacturer’s instructions. Visium libraries were analyzed via quantitative polymerase chain reaction (PCR) (QuantStudio 6, Applied Biosystems), and a total of 18 to 20 (standard workflow) or 12 to 14 (CytAssist workflow) PCR cycles were used for library amplification. Visium libraries were quantified with Qubit 1× double-stranded DNA high-sensitivity assay kit (Invitrogen, catalog no. Q33231) on a Qubit 4 Fluorometer (Invitrogen). Qualitative assessment of Visium libraries was conducted with the High-Sensitivity NGS Fragment Analysis Kit (Agilent, DNF-474) on a 96-channel fragment analyzer (Agilent) to assess size distribution and adapter dimer presence (<0.5%). All Visium libraries were normalized, pooled, and spiked with 10% PhiX Control v3 (Illumina), then loaded at 0.75 nM, and sequenced paired-end (Rd1: 28, Rd2: 10, Rd3: 10, and Rd4: 50) on a NovaSeq 6000 (Illumina).

### Xenium in situ

Xenium experiments were performed according to the manufacturer’s protocol using the predesigned human lung panel v1.1 able to identify 289 genes. Briefly, 5-μm sections were mounted on Xenium slides and went through incubations on a thermocycler for probe hybridization, ligation, and amplification. The run was processed with the instrument software version 1.6.1.0 and analysis version 1.6.0.8.

### Lunaphore COMET high-plex sequential immunofluorescence

FFPE tissue sections were baked for 1 hour at 60°C in an oven and then dewaxed and antigen-retrieved at pH 9 on a Leica BOND RX. Tissue sections were loaded onto the COMET platform and stained using a sequential immunofluorescence protocol according to the manufacturer’s instructions. Images were loaded into the Lunaphore Viewer, background-subtracted, and exported as ome.tiff files with each channel downsized to 8 bit. Image analysis was performed using Indica Labs HALO software.

Unconjugated antibodies (FOXJ1, clone EPR21874; CTHRC1, clone EPR22851-145; CD68, clone KP1; CD3, clone MRQ39; CD20, clone L26; CD66b, clone EPR25354-2; CD138, clone IHC138; LYVE1, clone EPR21857; VWF, clone D8L8G; AIF1, clone EPR16589; CLEC9A, clone EPR22324; KRT5, clone XM26; KRT17, clone E3; MMP7, clone EPR17888-101) were diluted in LI-COR Intercept (TBS) Blocking Buffer. Secondary anti-mouse Alexa Fluor Plus 555 and anti-rabbit Alexa Fluor Plus 647 antibodies (Thermo Fisher Scientific, A32727 and A32733) were diluted in LI-COR Intercept (TBS) Blocking Buffer at 1:200 and 1:400, respectively, with 4′,6-diamidino-2-phenylindole (Invitrogen/Thermo Fisher Scientific, D21490; 1 mg/ml) added as the counterstain at a dilution of 1:4000.

### Preprocessing of spatial Visium RNA-seq data

The Space Ranger computational pipeline from 10x Genomics was used to generate count matrices (v1.3.1 and v2.0 for CytAssist samples). The reads were aligned to the hg38 human reference genome (GRCh38-2020-A) and against the probe set references for human provided by 10x Genomics (v1 for FFPE samples and v2 for CytAssist samples). After preprocessing, analysis of the spatial Visium RNA-seq data was performed with the Python packages SCANPY (version 1.9.3) (*56*), Squidpy (1.2.3) (*62*, *63*), and cell2location (0.1.3) (*64*). The Space Ranger output files and the corresponding histology images were merged into a single anndata (*65*) object. Quality control parameters were assessed, and the data were filtered to remove spots with less than 800 counts, more than 45000 counts, less than 800 genes, or fewer than 25 cells. We log-transformed the data via SCANPY’s pp.log1p() function and calculated highly variable genes (top 6000 genes with flavor = seurat_v3). Using the highly variable genes as input, we used scvi-tools (*57*, *66*) to model the latent variables that were then used for Uniform Manifold Approximation and Projection and Leiden clustering, using 30 neighbors, which then was both transferred back to the full-gene dataset.

### Visium spot cell type deconvolution with cell2location and niche identification using NMF

For Visium spot deconvolution into cell types, we used cell2location and followed the tutorial provided on their GitHub (*64*). We loaded the PF-ILD atlas, filtered for IPF and control diagnosis, and performed permissive gene selection with standard settings before estimating reference cell type signatures with batch_key set to sampleID and the model training set to 250 epochs. Next, both the reference and the spatial input data were subsetted to shared genes, the cells per location parameter set to 8 and detection alpha to 20. The cell2location model was trained with 15,000 epochs and the 5% quantile of the estimated posterior distributions of cell abundance exported to the anndata object.

To identify cellular niches across tissue sections, we used the NMF function of the cell2location package on the predicted cell type abundances. We calculated 2 to 30 factors and found 9 factors to best represent cellular niches. While smaller numbers did not capture histological hallmarks such as airways, larger numbers resulted in “single cell–type factors.” The described niches are a compromise, representing histological hallmarks and changes in disease that could be given representative names. With the factor for artery-only scoring for a handful of cells, we combined it with the SMCs_Adv_Meso factor to end up with eight niches.

### Pseudo-Visium spots on Xenium data and deconvolution with cell2location

To assess accuracy of the cell2location spot deconvolution into cell type fractions, we grouped together cells on the Xenium sections in Visium-sized spots to form a pseudo-bulk transcriptomes. Next, we applied cell2location onto these pseudo-spots, as described above, to deconvolute them into cell type fractions. We correlated those predicted cell type fractions with the count of real single cell grouped into the pseudo-spots.

### Cell type frequency comparison

To compare the cell type frequencies between the modalities, we averaged the normalized cell type frequency per cell in the scRNA-seq data and the normalized estimated cell type frequency per spot in the spatial Visium data across the samples and diagnosis.

### Niche frequencies

To assess the impact of a cell type on the different niches, we calculated both the frequency of cell types within each niche and the normalized frequency of cell type contributions across all the niches, considering that a cell type could be rare but exclusively affect one niche.

### Gene pathway activity scoring

For each spot, we estimated signaling pathway activities with PROGENy’s (v1.12.0) weighted human model matrix using signature genes with a *P* value cutoff of 0.01. To infer pathway enrichment scores per spot, we ran the multivariate linear model from decoupleR (*24*) and summarized the scores across niches.

### Neighbor interactions

We first calculated a connectivity graph using the Squidpy’s gr.spatial_neighbors() method. This graph consists of cells (nodes) and cell-cell interactions (edges). Next, we wanted to identify niches that are spatially enriched for one another using a neighborhood enrichment test gr.nhood_enrichment(). Accessing the edges count, we normalized the interactions per niche and compared them between diagnoses by manually adding those niches that only exist in of the diagnosis. We extended this analysis, comparing immediate and extended niche neighborhood, by calculating the connectivity graphs for 1-ring or 3-rings of spots around a center target spot.

### GO and MSigDB enrichment

We used SCANPY’s tl.rank_genes_groups() to calculate niche specific marker genes using the Wilcoxcon test and filtered the results for genes to have a adjusted *P* value smaller than 5%, a log_2_ fold change greater than 0.5, be expressed within more than 25% of spots of a niche and less 25% outside the niche. Gene set enrichment analysis was then performed using GSEApy (1.0.0) (*67*) with the libraries “GO_Biological_Process_2021” and “MSigDB_Hallmark_2020” from the human database. Gene sets were considered enriched in the respective signature at a statistically significant level if the false discovery rate–corrected *P* value was below 0.01. Signatures were plotted in the heatmap if they were statistically significant in at least one niche.

### Gene signature scoring using hotspot

For scoring the senescence gene signature of the genes *GLB1*, *TP53*, *SERPINE1*, *CDKN1A*, *CDKN1B*, and *CDKN2B*, we used the compute_scores function from hotspot (*68*). Briefly, the counts for each gene are fit into the DANB model (depth-adjusted negative binomial), and the values for each gene are then scaled (centered) and then smoothed on the basis of neighbors of each cell. Last, these values for all genes in the signature are dimensionality reduced using principal components analysis. We used the log1p counts layer, 30 neighbors, and the X_scVI embedding to calculate the score.

### Cell-cell communication analysis of rebuilt spatial niches in scRNA-seq data

To analyze the spatially informed rebuilt cellular niches in the scRNA-seq data, we used CellChat (*69*). We created the CellChat database with the human interactions. The CellChat objects were created from the PF-ILD atlas subsetted to only the IPF and control diagnosis samples and were run separately per condition, before being combined for the analysis. The niches were rebuilt by selecting the cell types that were overrepresented in the NMF-derived niches/factors (Fig. 2A) and subsetting the CellChat object accordingly. The interactions per niche were filtered for a *P* value smaller than 0.01. For a better representation, we split the analysis and figure panels based on the CellChatDB annotation categories that group the interactions into cell-cell contact, secreted signaling, and ECM-receptor.
