# Supplementary material for: Spatial transcriptomic characterization of pathologic niches in IPF
Source: Sci Adv. 2024 Aug 9;10(32):eadl5473. doi: 10.1126/sciadv.adl5473 (PMC11313858; doi:10.1126/sciadv.adl5473)
Supplement: Supplementary file 1 — Figs. S1 to S20 Tables S2 and S7 Legends for tables S1, S3 to S6 [file sciadv.adl5473_sm.pdf]

Science Advances
AAAS

Supplementary Materials for

Spatial transcriptomic characterization of pathologic niches in IPF

Christoph H. Mayr *et al.*

Corresponding author: Christoph H. Mayr, christoph.mayr@boehringer-ingelheim.com;
Fidel Ramirez, fidel.ramirez@boehringer-ingelheim.com;
Matthew J. Thomas, matthew_james.thomas@boehringer-ingelheim.com

Sci. Adv. **10**, eadl5473 (2024)
DOI: 10.1126/sciadv.adl5473

The PDF file includes:

Figs. S1 to S20
Tables S2 and S7
Legends for tables S1 and S3 to S6

Other Supplementary Material for this manuscript includes the following:

Tables S1 and S3 to S6

Supplemental Figures

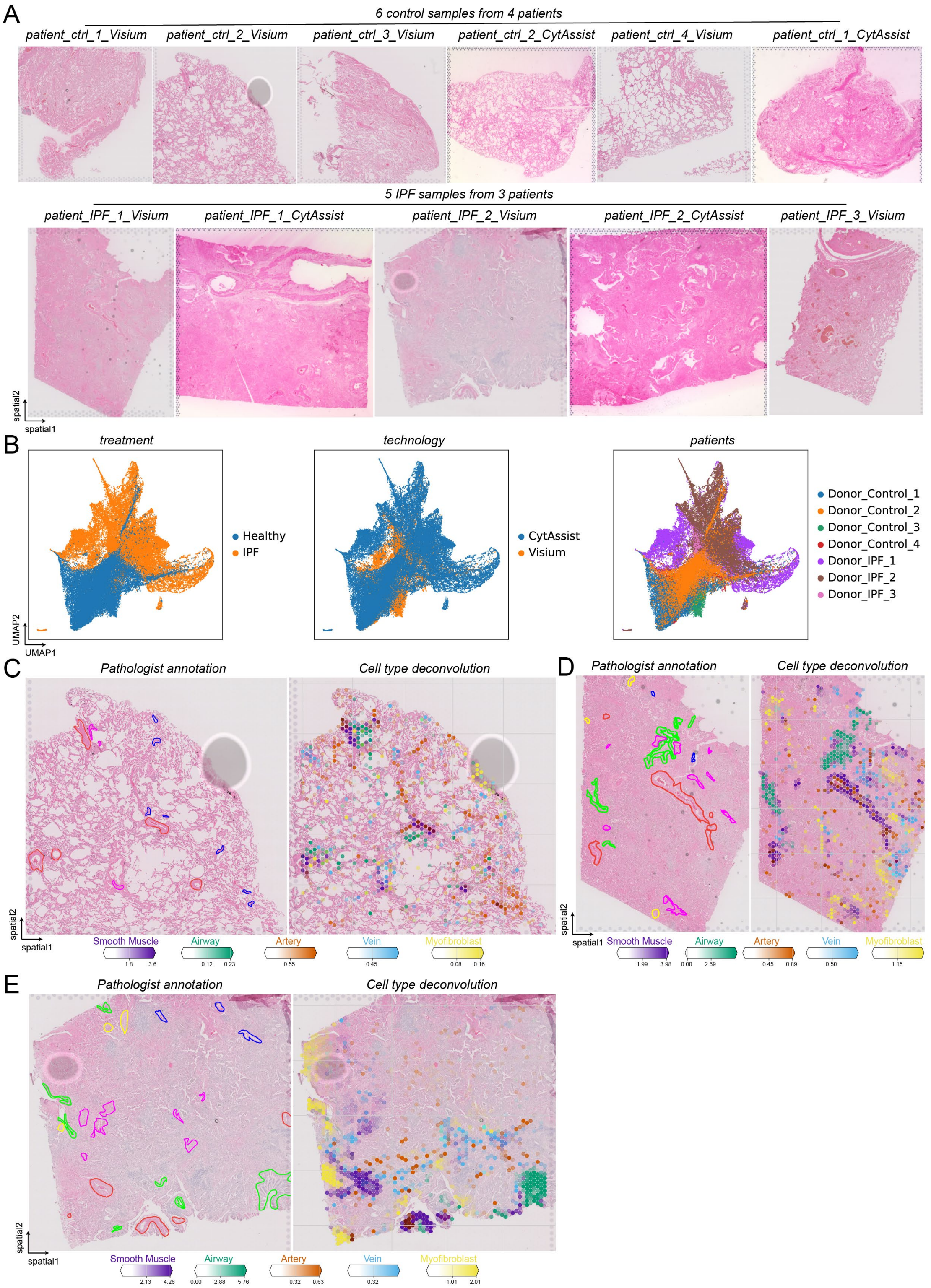

Figure S1: Overview of tissue sections used for Visium for FFPE and CytAssist assays.
(A) H&E-stained tissue sections of IPF and control patient lungs, used for the indicated assays of either 10x Genomics Visium for FFPE or 10x Genomics CytAssist for FFPE, or both. **(B)** The integrated spatial data of all tissue section is visualized as UMAP. The color code illustrates the disease status, the different technologies, and the donors. **(C-D)** The plots show the comparison of tissue annotated by a pathologist and the respective predicted cell type abundance from cell2location after spot deconvolution for **(C)** a control section and **(D-E)** two IPF sections.

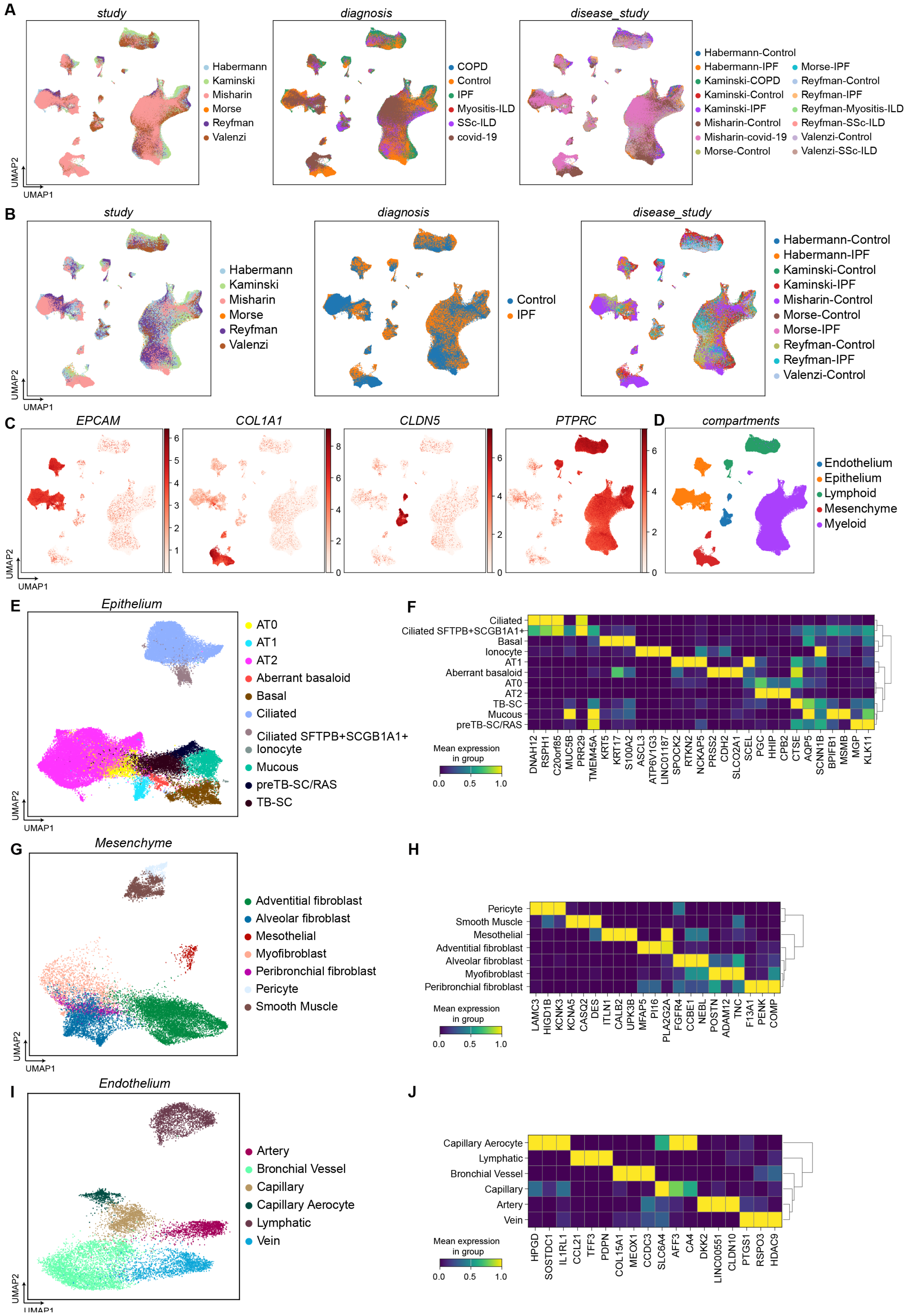

Figure S2: Overview of PF-ILD scRNAseq atlas data and description of compartments.

(A) The integrated scRNAseq data of the PF-ILD atlas is visualized through uniform manifold approximation and projection (UMAP). The color code illustrates the different studies/datasets, diagnosis, and their combination. **(B)** UMAPs represent the reduced atlas dataset to only IPF and control diagnosis. The color code illustrates the different studies/datasets, diagnosis, and their combination. **(C-D)** The indicated marker genes were used to select clusters for subsetting the data into compartments. **(E-J)** The compartments are represented with their respective UMAPs and top 2 marker genes per cell type within the compartment, for **(E-F)** *EPCAM*+ Epithelial cells, **(G-H)** *COL1A1*+ mesenchymal cells, **(I-J)** *CLDN5*+ endothelial cells.

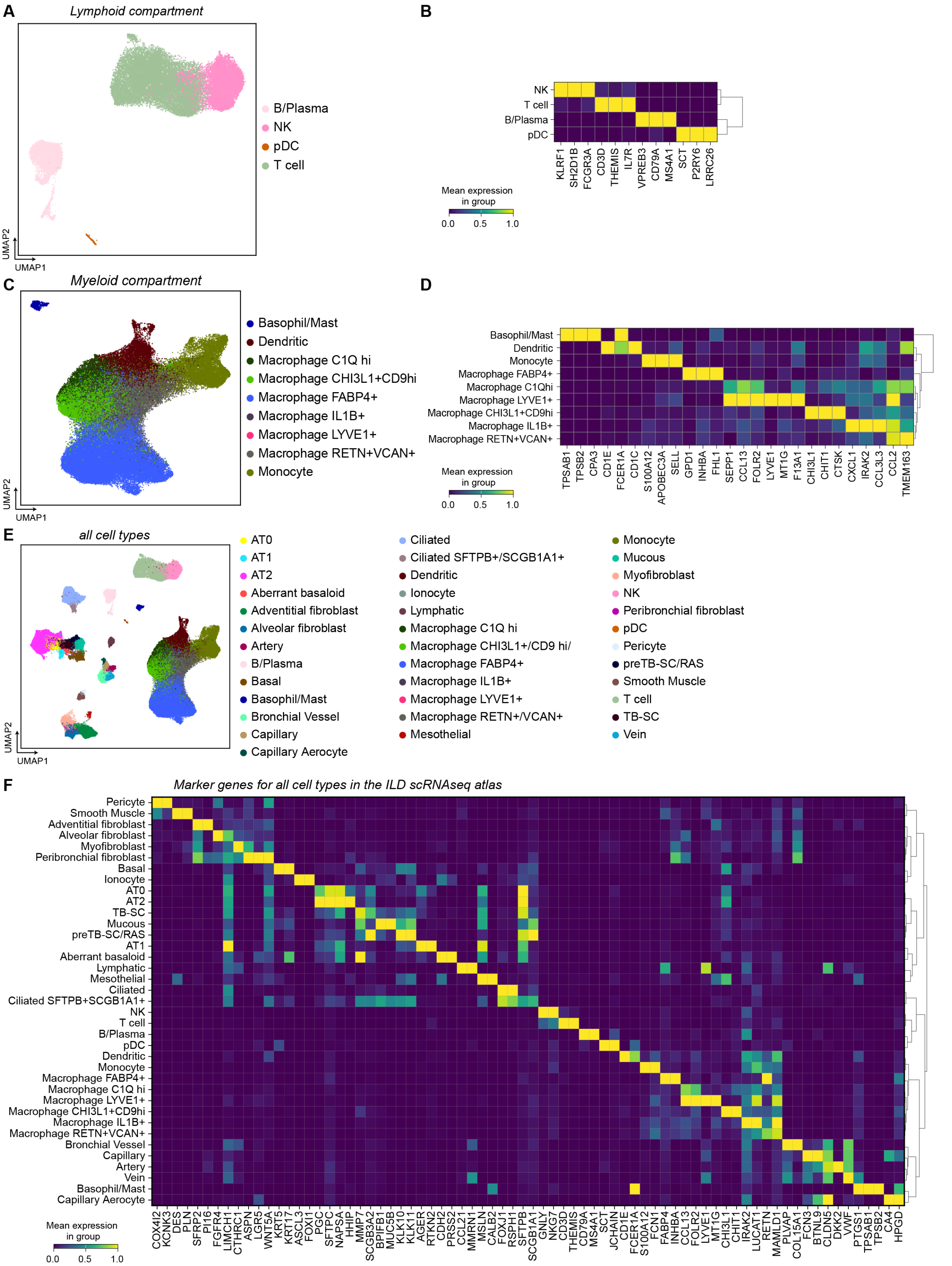

Figure S3: Second part of Overview of scRNAseq atlas data and description of compartments.

(A-D) The compartments are represented with their respective UMAPs and top 2 marker genes per cell type within the compartment, for **(A-B)** Lymphoid cells, **(C-D)** Myeloid cells. **(E)** The color code of the UMAP indicates all annotated cell types. **(F)** The heatmap shows normalized mean expression for accepted cell type marker genes per cell type.

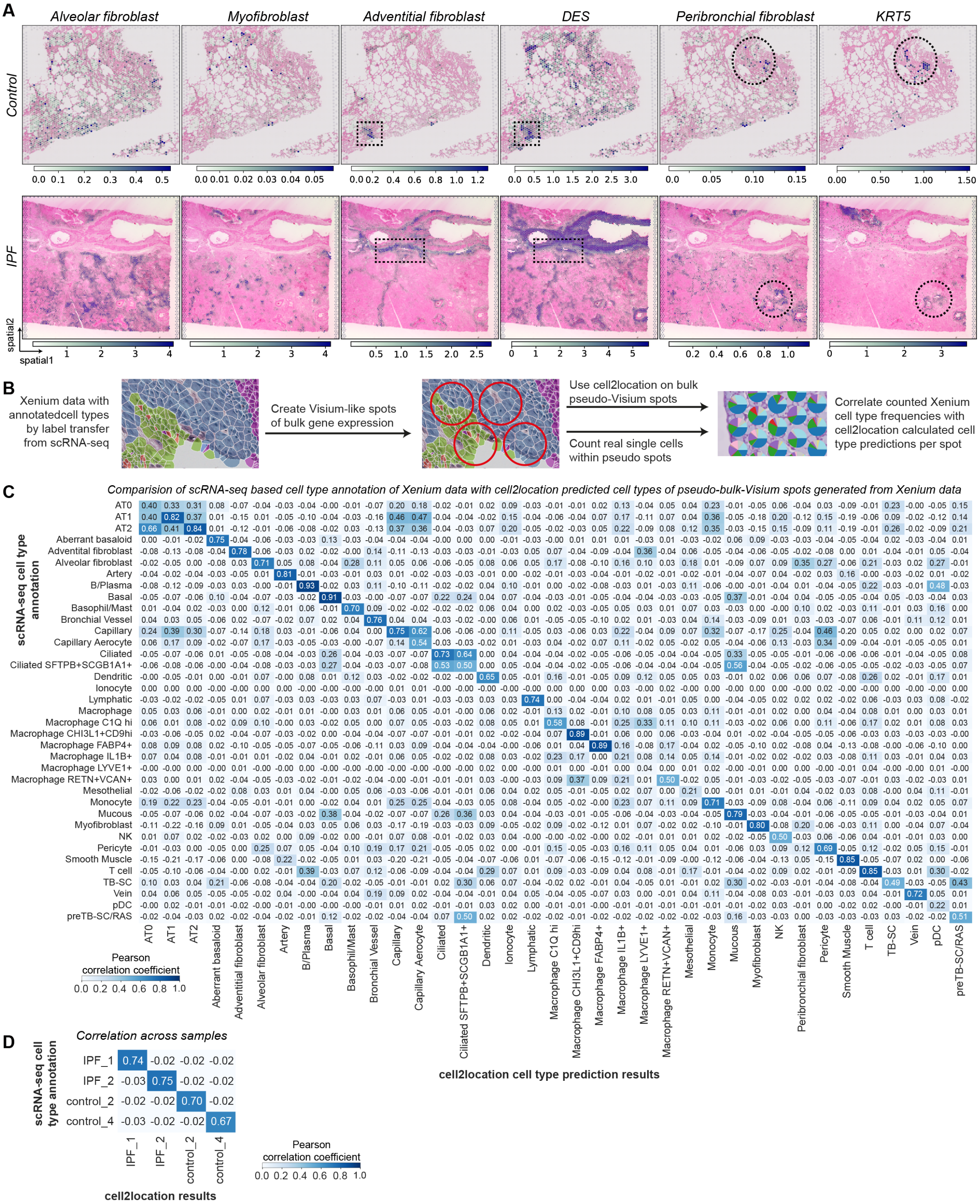

Figure S4: Validation of cell2locatio predicted cell types.

(A) Spatial plots exemplary show one control and IPF section with cell2location predicted fibroblast subtype cell type abundances. Desmin (*DES*) is shown as a marker for blood vessels and Keratin 5 (*KRT5*) as a marker for airways. **(B)** The cartoon illustrates on how the comparison of real single cells from Xenium data with predicted cell2location cell type abundances were performed, applying pseudo Visium spots on the Xenium data. **(C)** Heatmap displays the Pearson correlation of real cell types in pseudo Visium spots in the Xenium data versus cell2location predicted cell type abundances in those pseudo spots. **(D)** Heatmap shows the same correlation as in (C) but across samples.

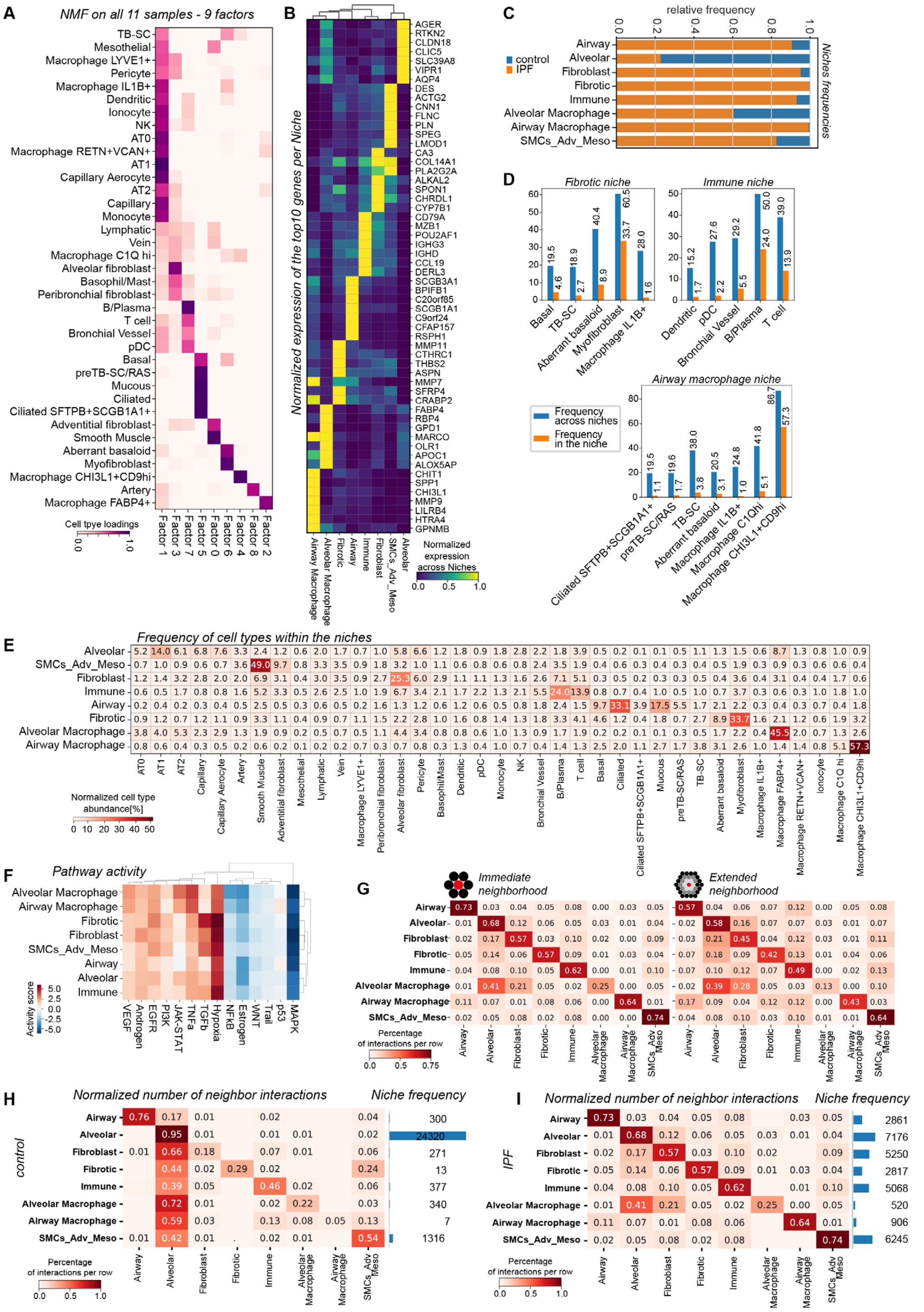

Figure S5: Characterization of NMF inferred cellular niches. (A) The heatmap shows normalized cell type abundance for each of the 9 factors as calculated by the non-matrix factorization (NMF) of the cell2location package. Factor 8 only scored for 12 spots and was combined with Factor 0 into the SMCs_Adv_Meso niche. **(B)** The heatmap shows normalized expression of top10 genes per niche as result of the allmarkers() function from scanpy(*55*). **(C)** The bar graph illustrated relative frequencies of the niches in IPF or control sections. **(D)** The bar graphs illustrate the distribution of total cell type frequency across all niches (bluE), and the contribution of cell types to one niche as percentage of all cell types in one niche (orangE), for the three disease associated niches. **(E)** The heatmap shows the composition of all niches as percentages of cell types. **(F)** The heatmap shows the pathway activity score per spot, summarized per niche. The R package decoupleR(*66*) was used to score the pathway activity as provided by the R package PROGENy(*66*). **(G)** The heatmap shows the interactions between the niches as percentage per row, calculated by the function spatial_neighbors() in squidpy(*61*) for the direct neighbors (1 ring of spots) or the externed neighborhood (3 rings of spots). **(H-I)** Heatmaps show normalized neighbor interactions for each niche in **(H)** control and **(I)** IPF tissue.

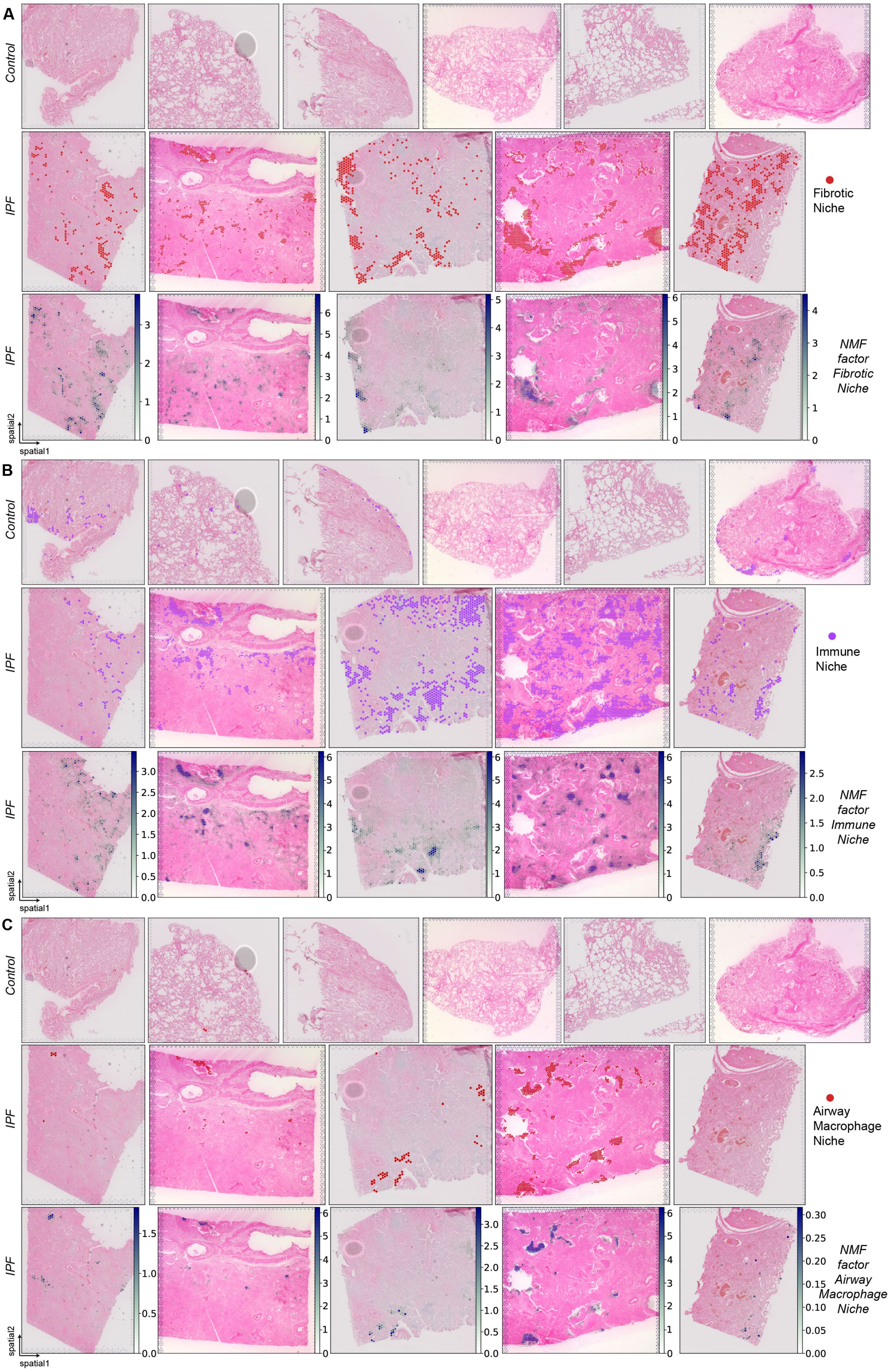

Figure S6: Pathological cellular niches across all tissue sections.

(A-C) The spatial plots display the distribution of the three in IPF emerging pathological cell type niches across all tissue sections, as well as the numeric NMF factor that was used for the assignment of spots to a niche, for **(A)** the fibrotic niche, **(B)** the immune niche and **(C)** the airway macrophage niche.

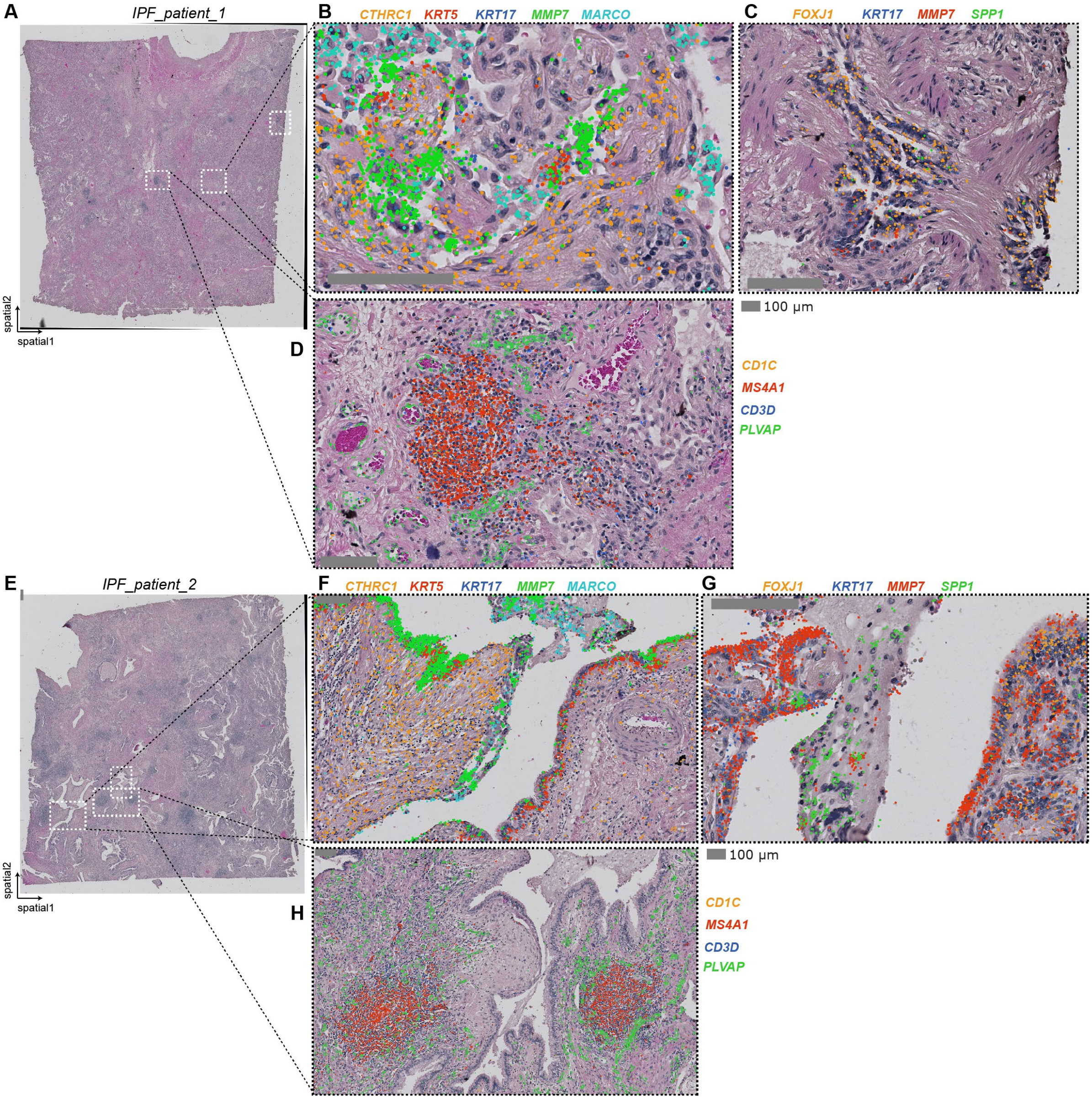

Figure S7: Validation of IPF associated niches in adjacent tissue sections of Visium samples.
(A, E) H&E images of adjacent tissue sections of **(A)** IPF_patient_1 and **(E)** IPF_patient_2. **(B-H)** Xenium mRNA in situ hybridization data for marker genes of respective cell types in the **(B, F)** fibrotic niche, with co-localization of *KRT5-KRT17+* aberrant basaloid cells with *CTHRC1+* myofibroblasts next to *KRT5+KRT17+* basal cells and *MMP7+* airway cells, **(C, G)** airway macrophage niche with co-localization of *SPP1+* macrophages inside the airway lumen, lined with *FOXJ1+* ciliated cells, *KRT17+* TB-SCs and *MMP7+* airway cells and **(D, H)** immune niche with *MS4A1* for B and plasma cells, *CD3D* for T cells and *CD1C* for dendritic cells within a distinct foci, surrounded by *PLVAP* for ectopic endothelial bronchial vessels.

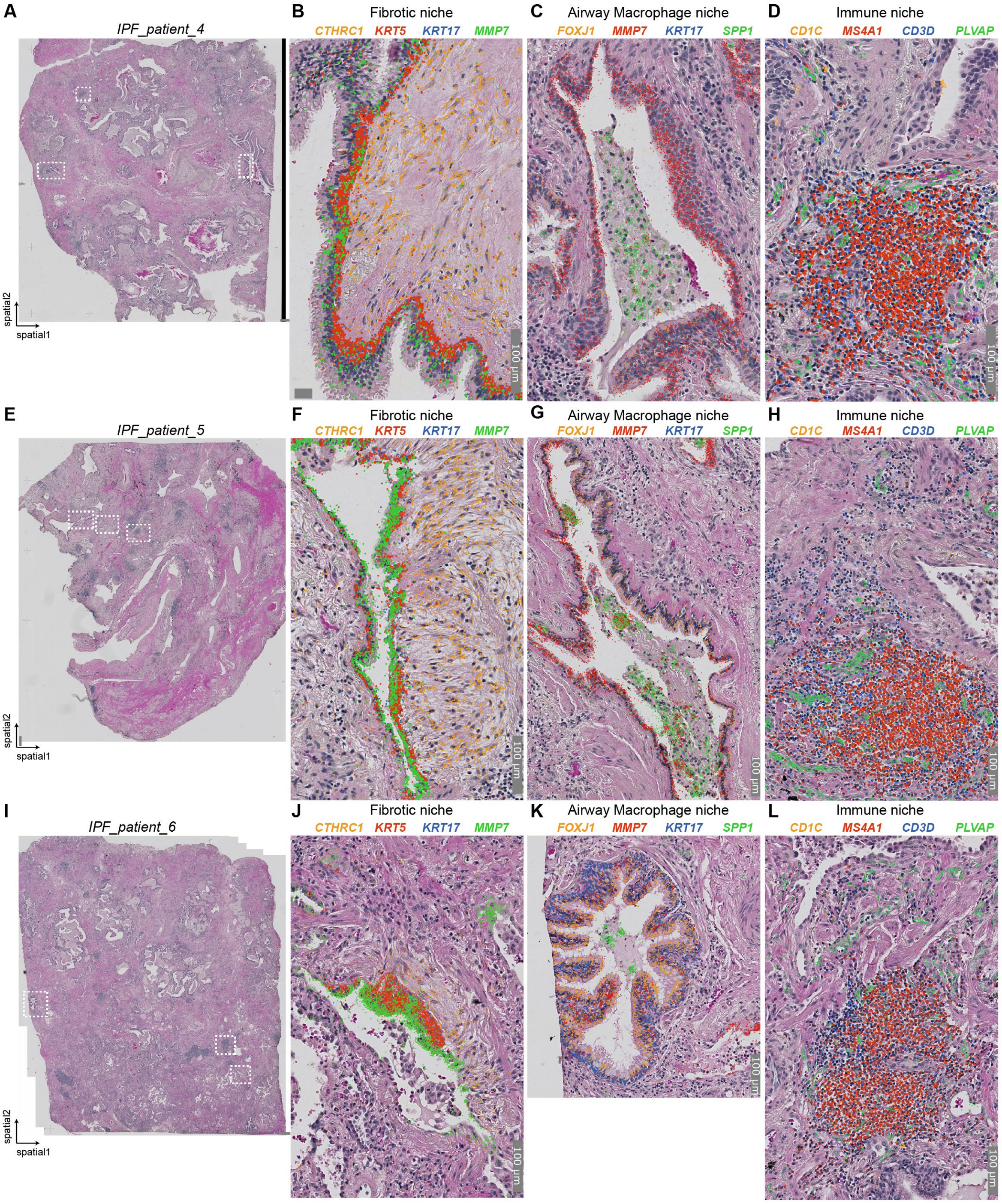

Figure S8: Xenium validation of IPF associated niches in additional IPF patient tissue sections.
(A, E, I) H&E images of additional tissue sections of **(A)** IPF_patient_4, **(E)** IPF_patient_5 and **(I)** IPF_patient_6. **(B-L)** Xenium mRNA in situ hybridization data for marker genes of respective cell types in the **(B, F, J)** fibrotic niche, with co-localization of *KRT5-KRT17*+ aberrant basaloid cells with *CTHRC1*+ myofibroblasts next to *KRT5*+*KRT17*+ basal cells and MMP7+ airway cells, **(C, G, K)** airway macrophage niche with co-localization of *SPP1*+ macrophages inside the airway lumen, lined with *FOXJ1*+ ciliated cells, *KRT17*+ TB-SCs and MMP7+ airway cells and **(D, H, L)** immune niche with *MS4A1* for B and plasma cells, *CD3D* for T cells and *CD1C* for dendritic cells within a distinct foci, surrounded by *PLVAP* for ectopic endothelial bronchial vessels.

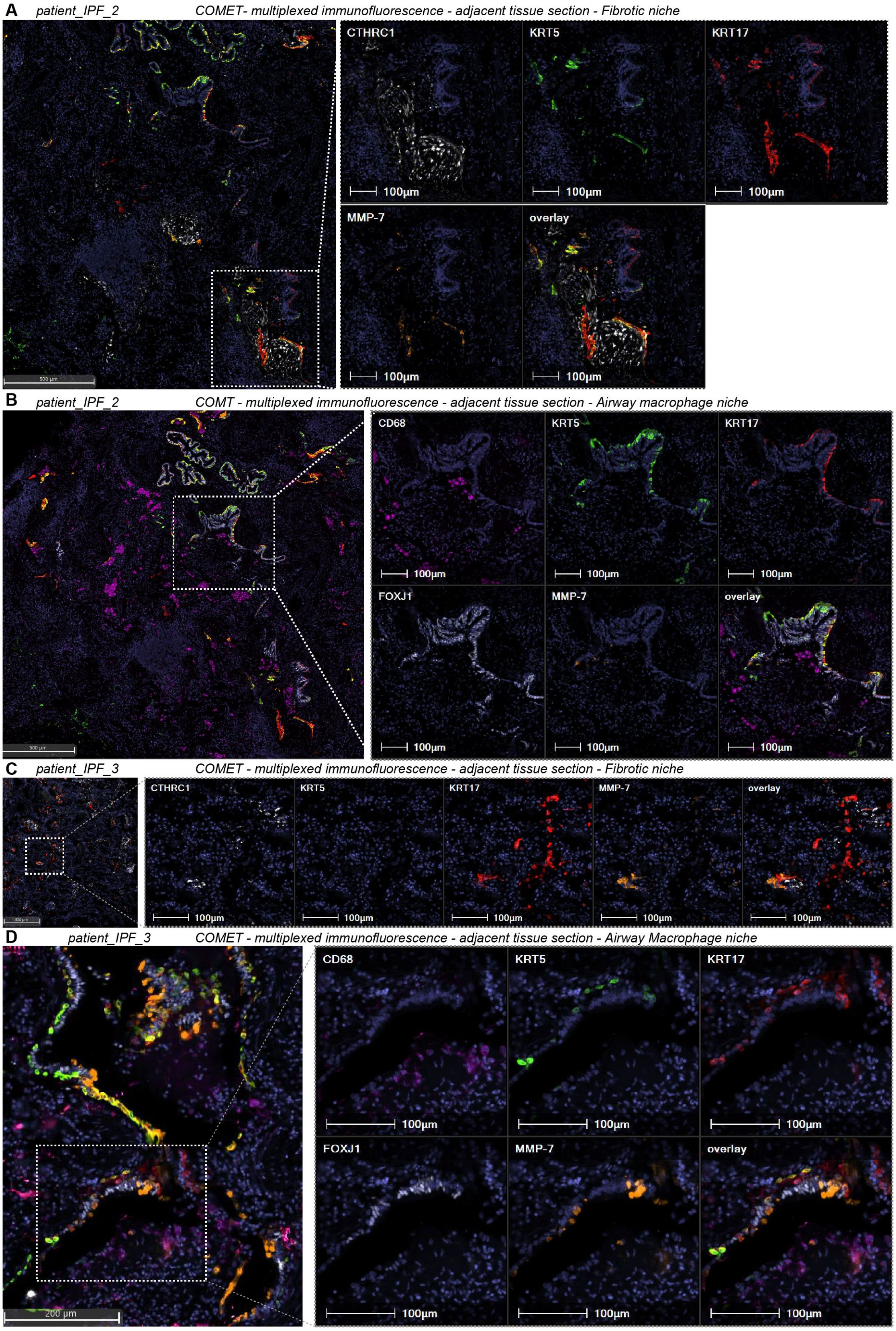

Figure S9: Protein validation of IPF associated niches on adjacent tissue sections of Visium samples. (A, C) COMET high-plex sequential protein immunofluorescence on adjacent tissue section showing the fibrotic niche with co-localization of KRT5-KRT17+MMP7+ aberrant basaloid cells with CTHRC1+ myofibroblasts next to KRT5+KRT17+ basal cells and MMP7+ airway cells and **(B, D)** COMET sequential protein immunofluorescence on adjacent tissue section showing the airway macrophage niche with airways lined with FOXJ1 for ciliated cells, KRT17 for TB-SC and MMP7 for airway cells and the airway lumen filled with CD68+ macrophages.

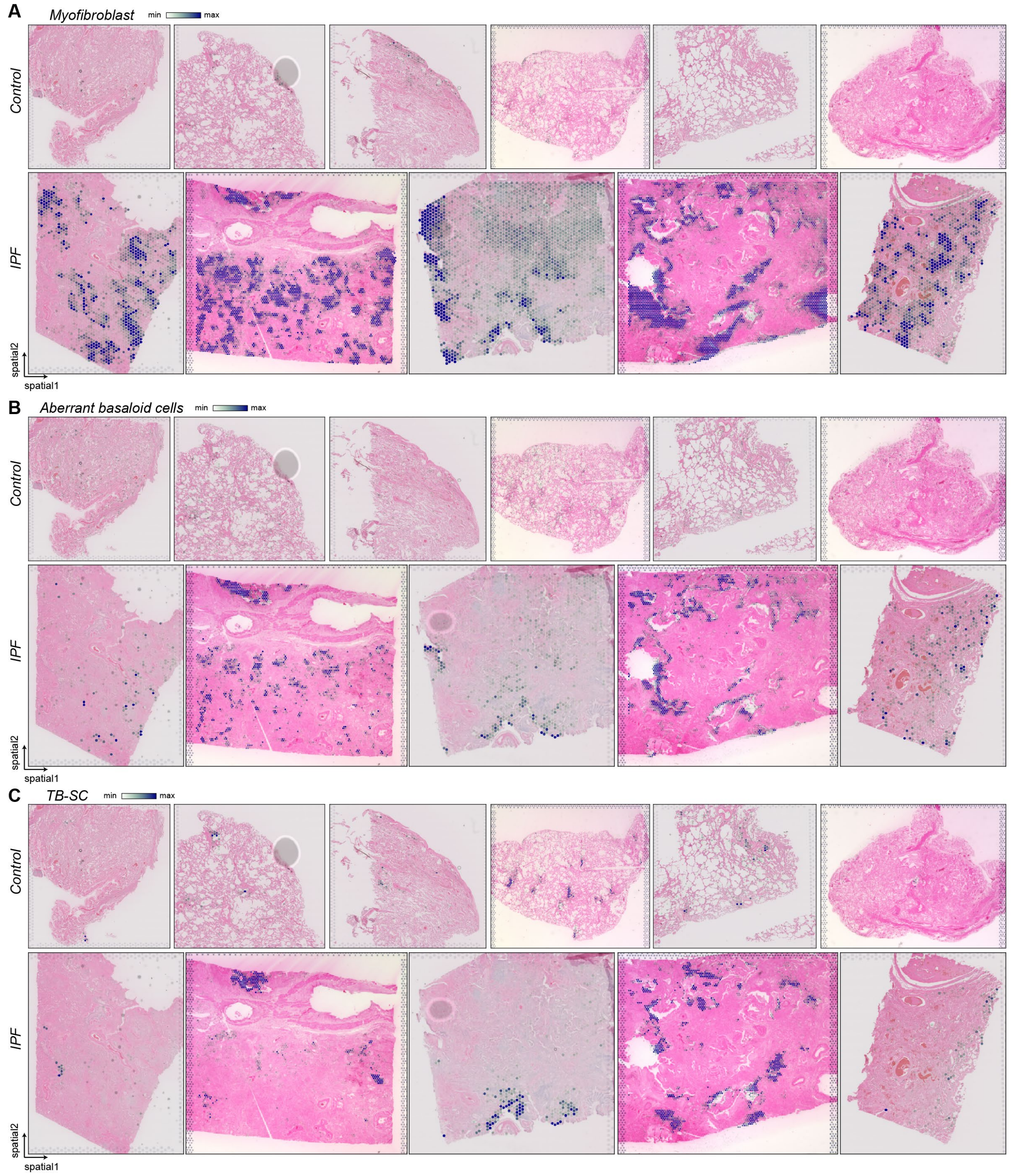

Figure S10: Localization of Fibrotic niche associated cell types.
(A-C) The spatial plots display cell type abundance across all tissue sections for **(A)** Myofibroblasts, **(B)** Aberrant basaloid cells, and **(C)** TB-SCs.

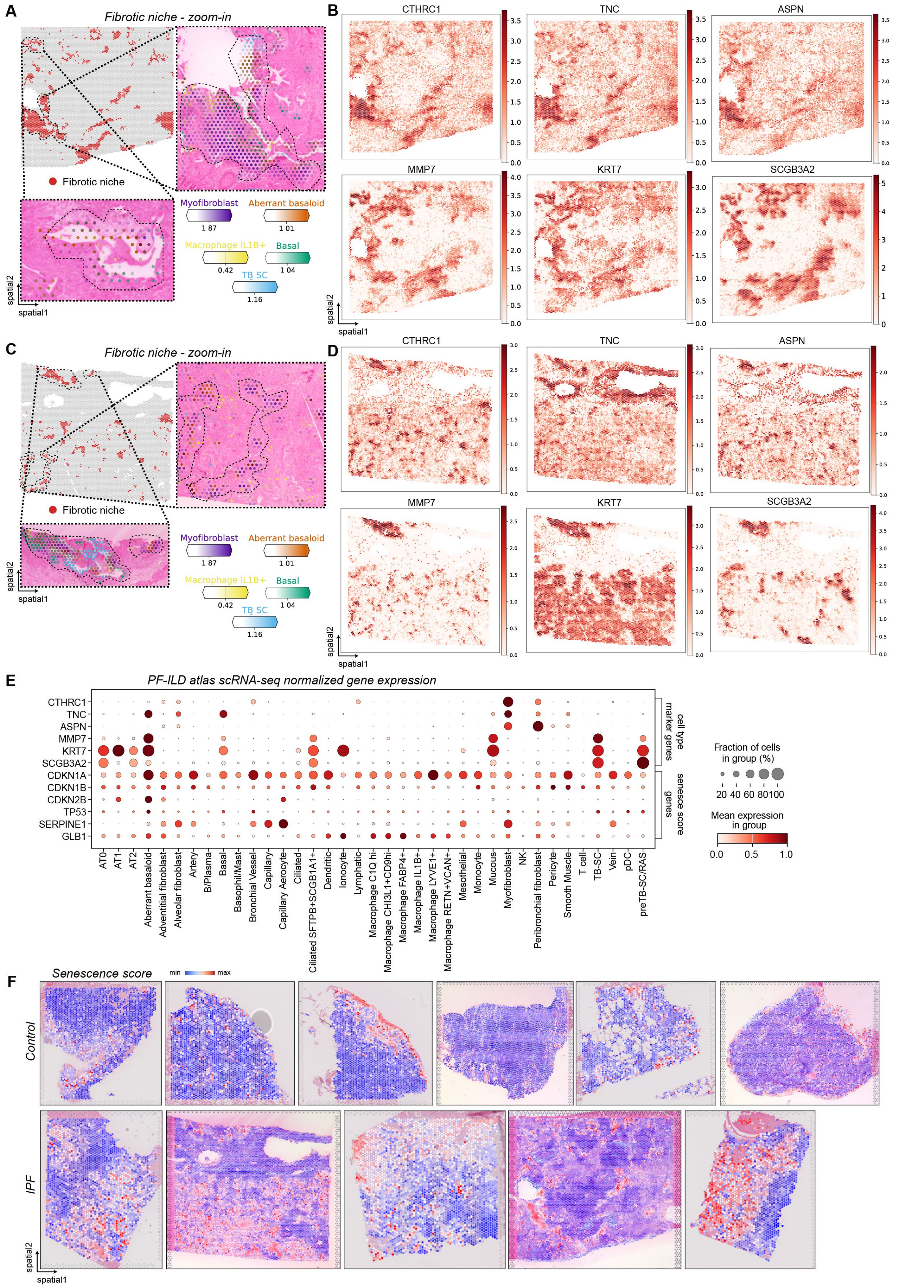

Figure S11: Localization and characterization of fibrotic niche. (A-D) For two IPF tissue sections, the spatial plots display **(A, C)** the distribution of the fibrotic niche across the slides and zoom ins of two example regions with of selected cell type abundances, as well as **(B, D)** indicated marker gene expression. **(E)** The dotplot displays gene expression across cell types in the IPF single cell atlas. **(F)** Senescence gene score (*CDKN1A, CDKN1B, CDKN2B, TP53, SERPINE1, GLB1*) calculated using hotspot gene-module scoring(*22*), across all tissue sections.

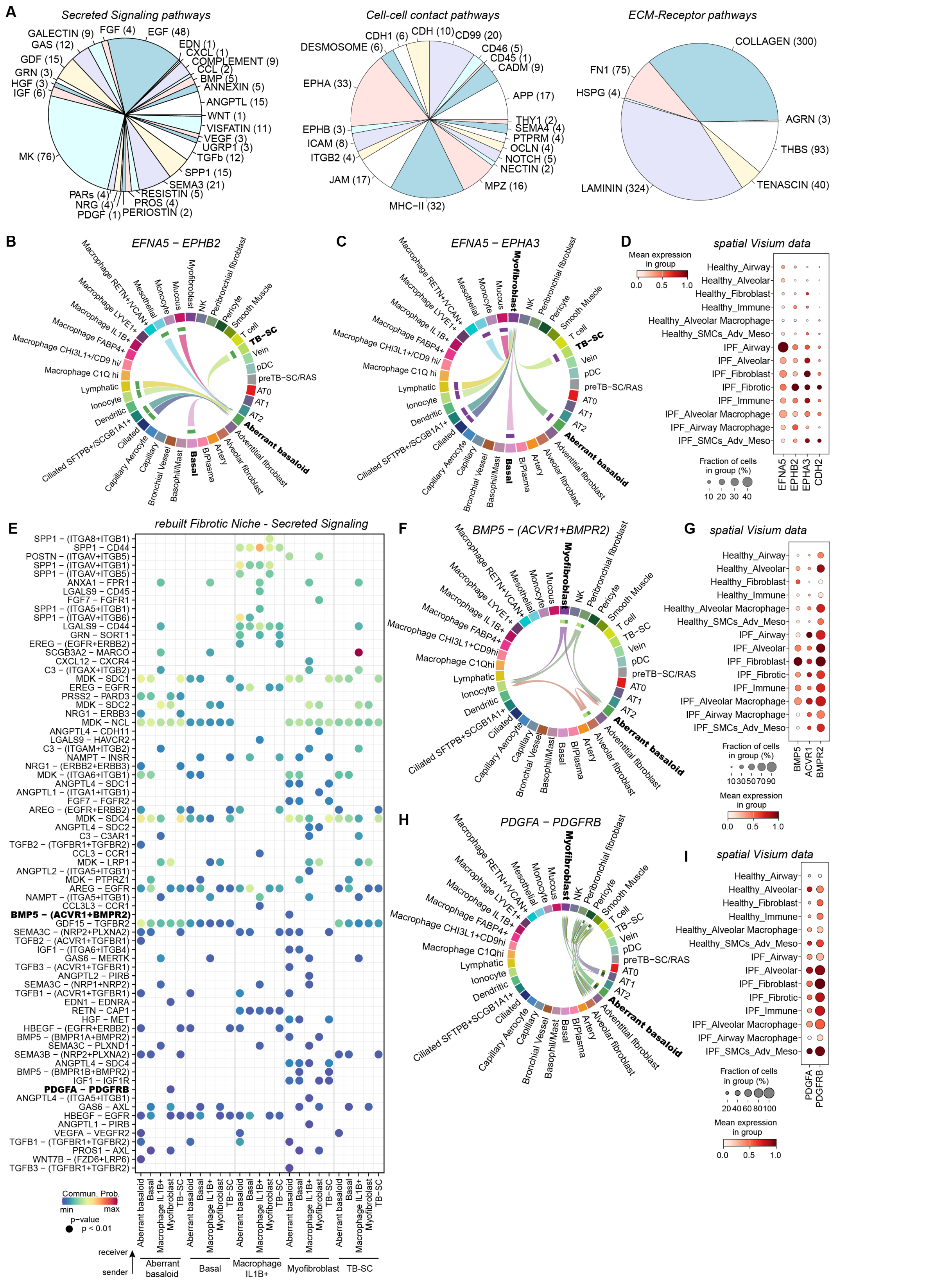

Figure S12: Cell-cell signaling within the fibrotic niche. (A) Pie charts show the distribution of receptor-ligand pairs into the three cell communication categories and their associated signaling pathways. **(B-C)** Chord plot visualizing the interacting cell types for the ligand-receptor pair **(B)** *EFNA5-EPHB2* and **(C)** *EFNA5-EPHA3*. **(D)** Gene expression of receptor and ligand genes across the niches and disease in the spatial Visium data. **(E)** Heatmap of significant ligand-receptor pairs from the secreted signaling category within the Fibrotic niche. **(F-I)** Chord plot visualizing the interacting cell types and heatmaps the expression of the involved genes in the spatial data for the ligand-receptor pairs **(F, G)** *BMP5-(ACVR1+BMPR2)* and **(H, I)** *PDGFA-PDGFRB*.

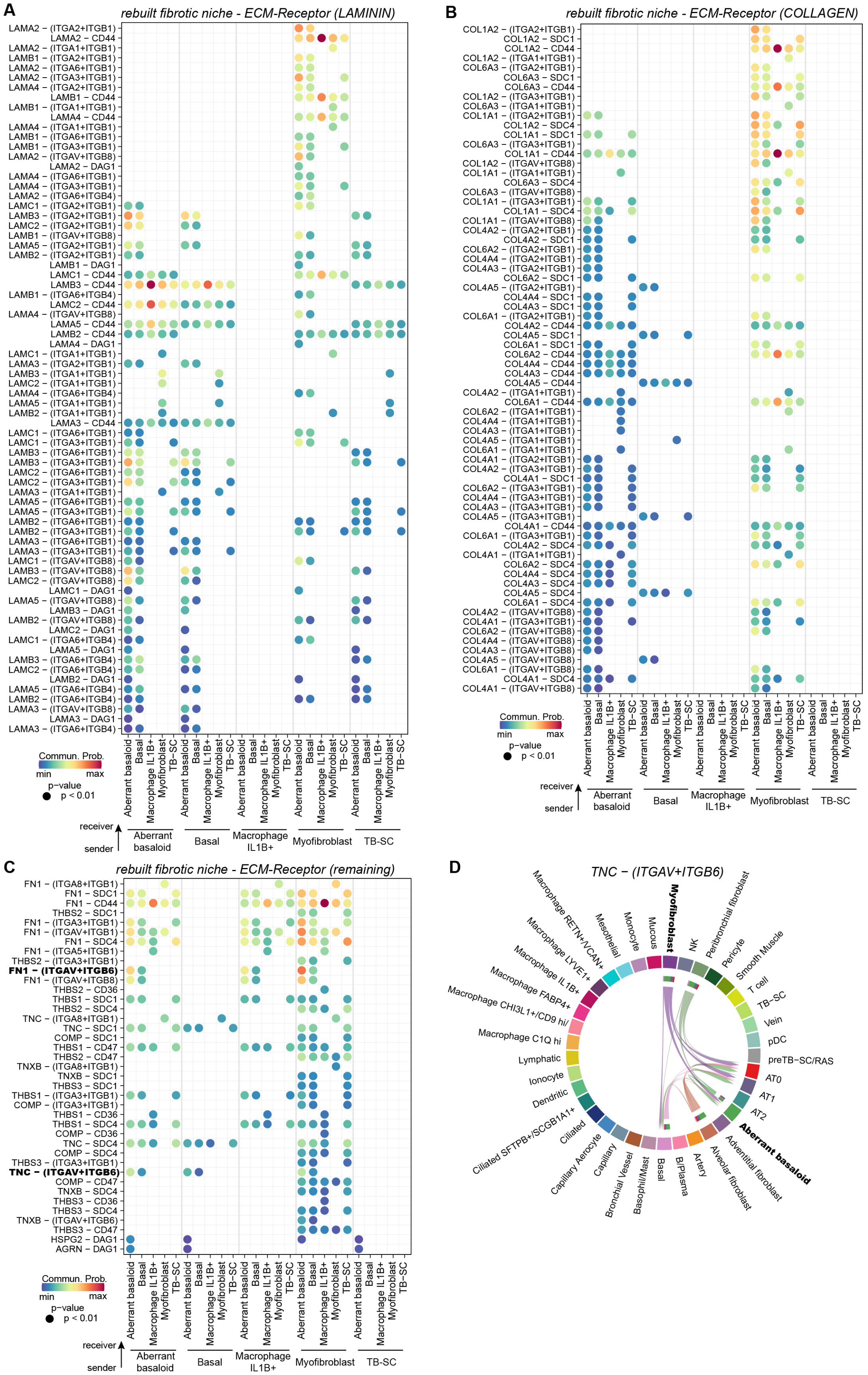

Figure S13: ECM-Receptor signaling within fibrotic niche.

(A-C) Heatmaps of significant ligand-receptor pairs from the ECM-Receptor category within the airway fibrotic niche, split into major pathways **(A)** laminin pairs, **(B)** collagen pairs and **(C)** the remaining pairs. **(D)** Chord plot visualizing the interacting cell types for the ligand-receptor pair *TNC-(ITGAV+ITGB6).*

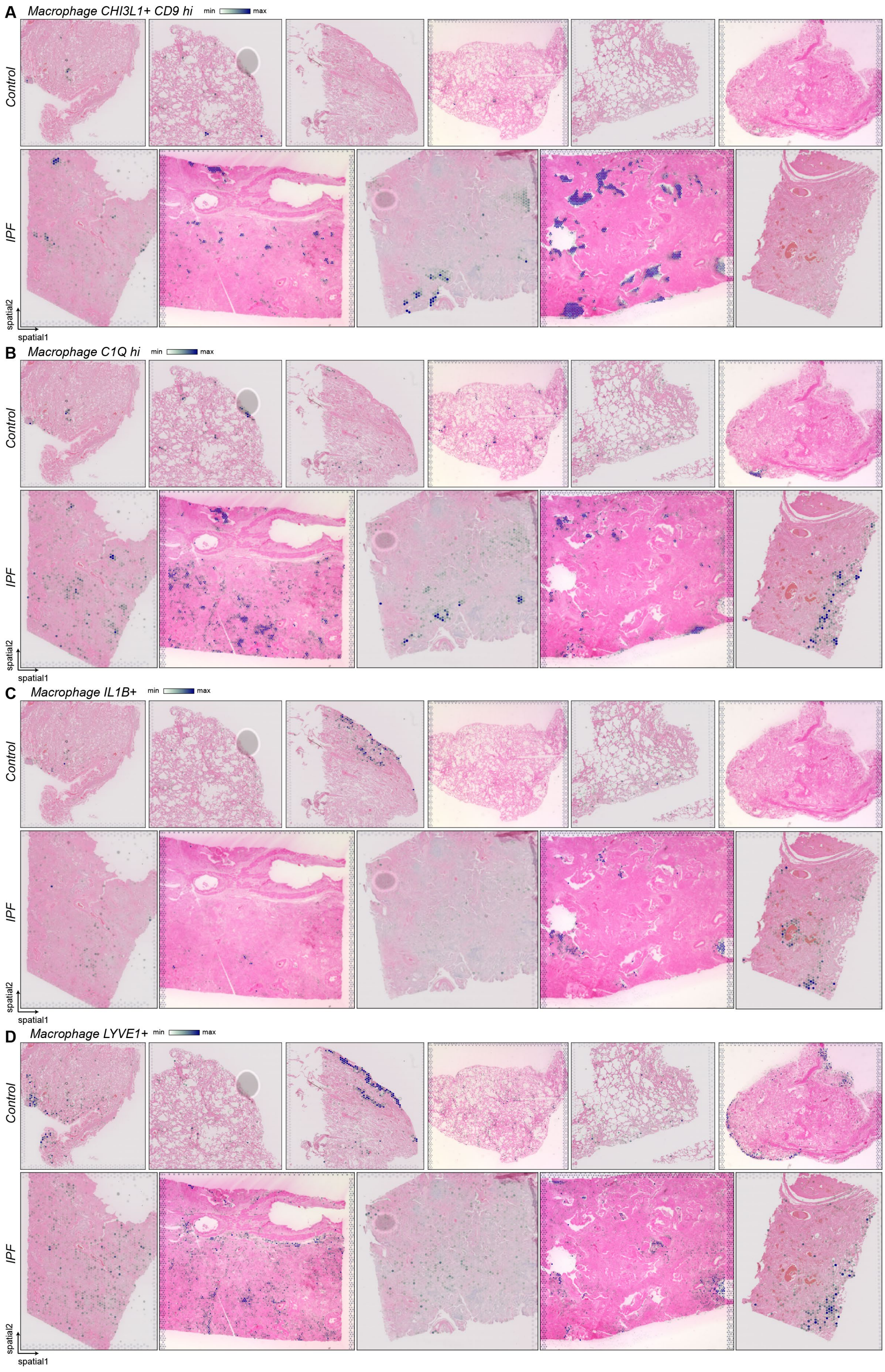

Figure S14: Localization of disease induced macrophage cell types.

(A-D) The spatial plots display cell type abundance across sections for the different macrophage cell subtypes of **(A)** *CHI3L1+ CD9hi* macrophages, **(B)** *C1Qhi* macrophages, **(C)** *IL1B+* macrophages and **(D)** *LYVE1+* macrophages.

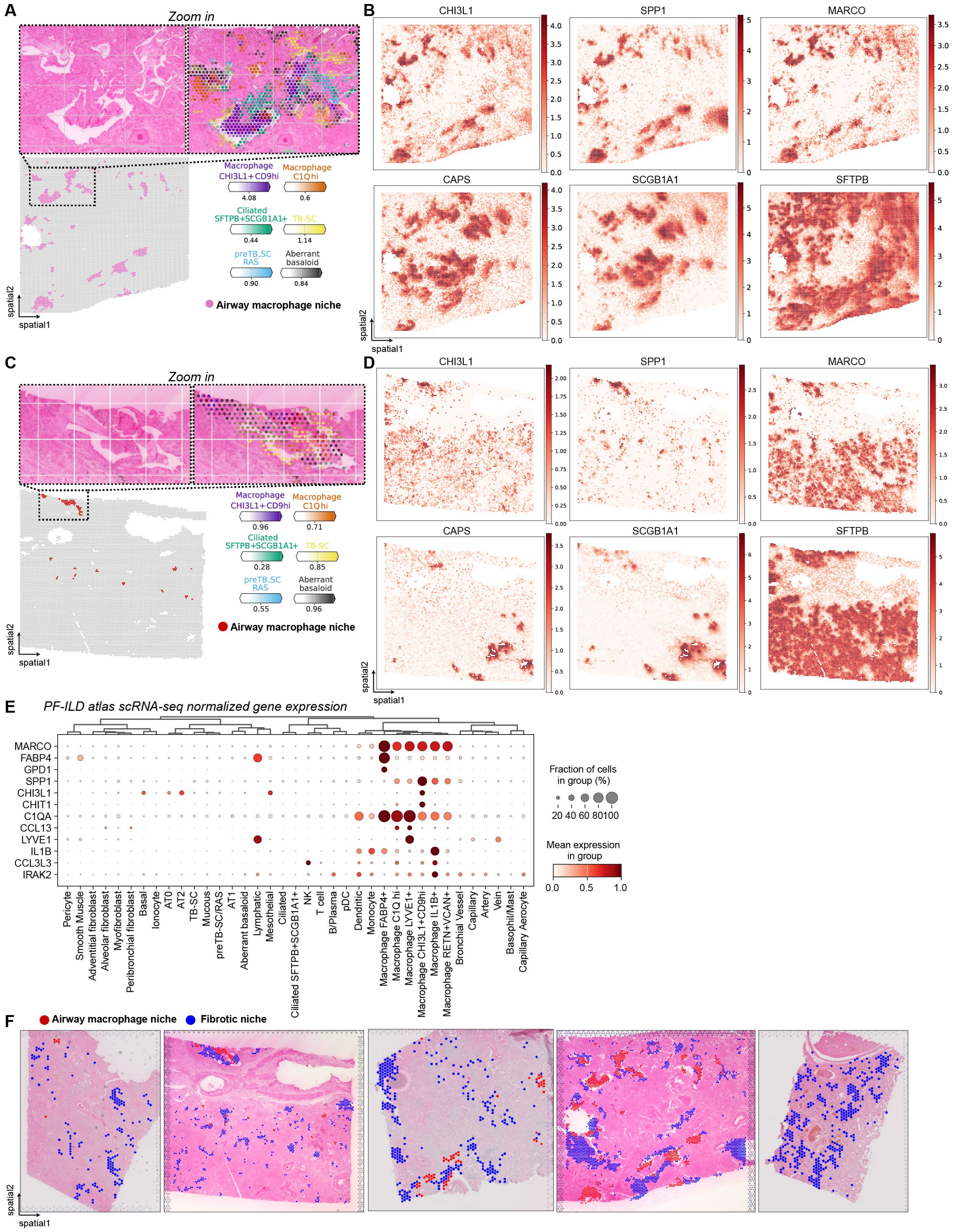

Figure S15: Localization and characterization of the airway macrophage niche.

(A-D) For two IPF tissue sections, the spatial plots display **(A, C)** the distribution of the airway macrophage niche across the slides and zoom ins of two example regions with of selected cell type abundances, as well as **(B, D)** indicated marker gene expression. **(E)** The dotplot displays indicated gene expression across cell types in the IPF single cell atlas. **(F)** The spatial plots show localization of the airway macrophage niche and the fibrotic niche across the 5 IPF tissue sections.

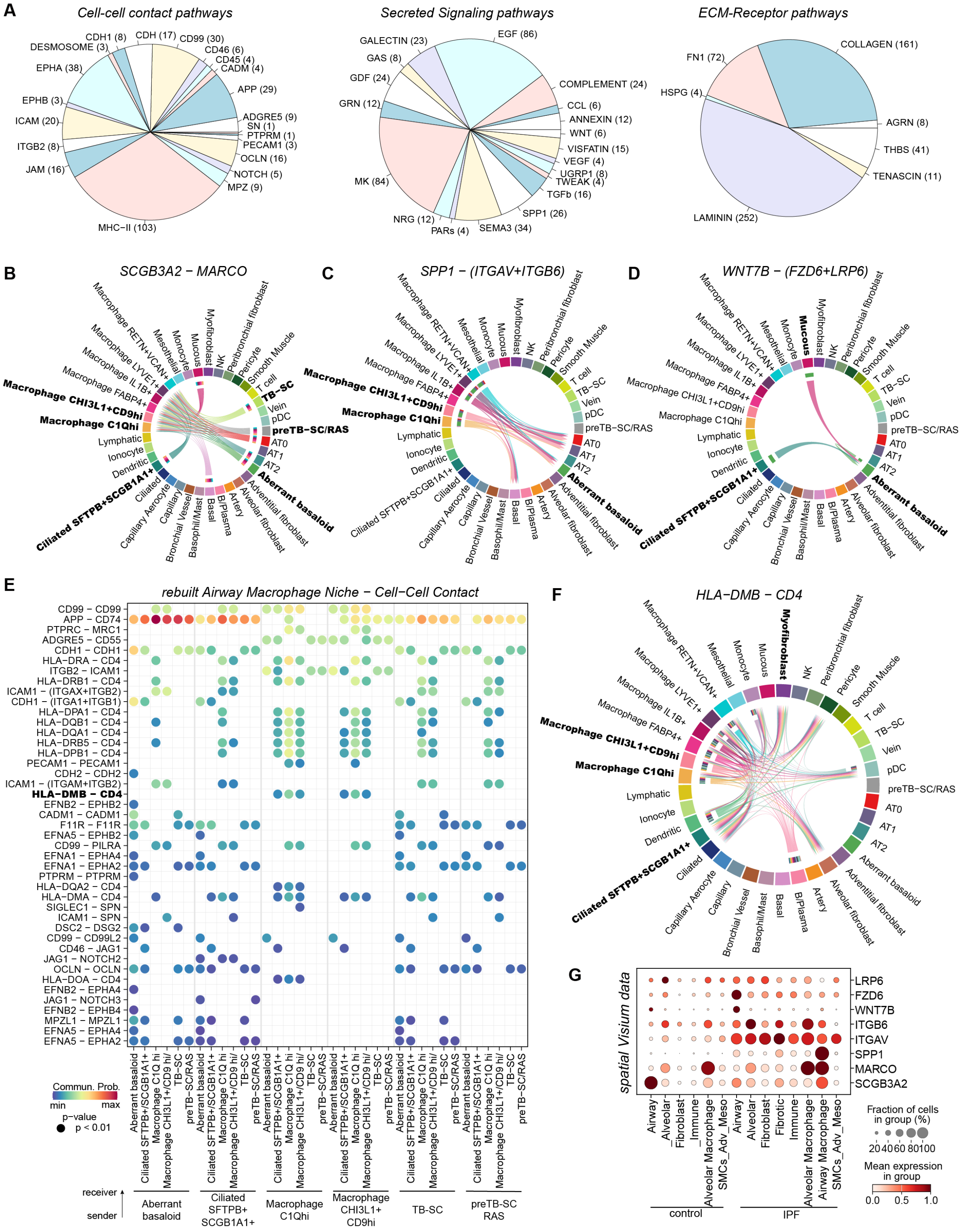

Figure S16: Cell-cell signaling within the airway macrophage niche.

(A) Pie charts show the distribution of receptor-ligand pairs in the airway macrophage niche into the three cell communication categories and their associated signaling pathways. **(B-D)** Chord plot visualizing the interacting cell types for the ligand-receptor pair **(B)** *SCGB3A2-MARCO*, **(C)** *SPP1-(ITGAV+ITGB6)* and **(D)** *WNT7B-(FZD6+LRP6)*. **(E)** Heatmap of significant ligand-receptor pairs from the cell-cell contact category within the airway macrophage niche. **(F)** Chord plot visualizing the interacting cell for the ligand-receptor pair *HLA-DMB – CD4*. **(G)** Gene expression of given receptor and ligand genes across the niche and disease in the spatial Visium data.

A

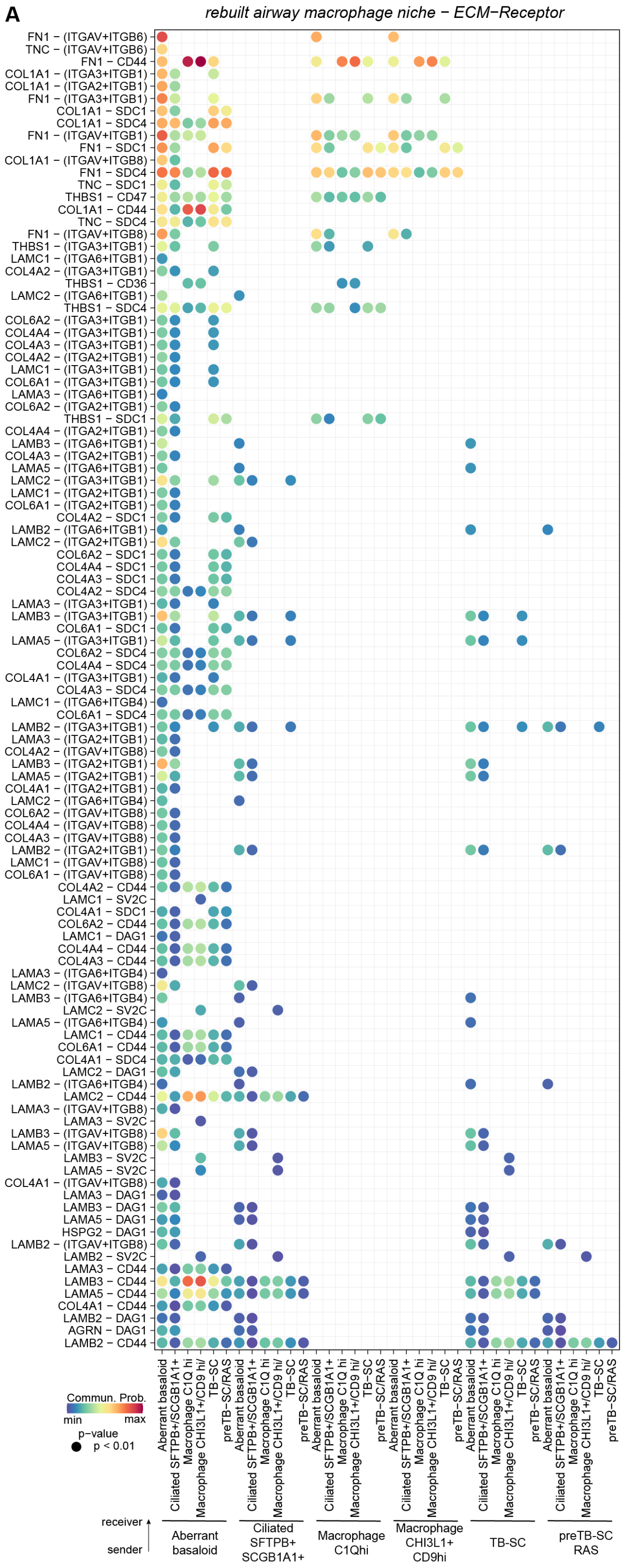

Figure S17: ECM-Receptor signaling within airway macrophage niche.
(A) Heatmap of significant ligand-receptor pairs from the ECM-Receptor category within the airway macrophage niche.

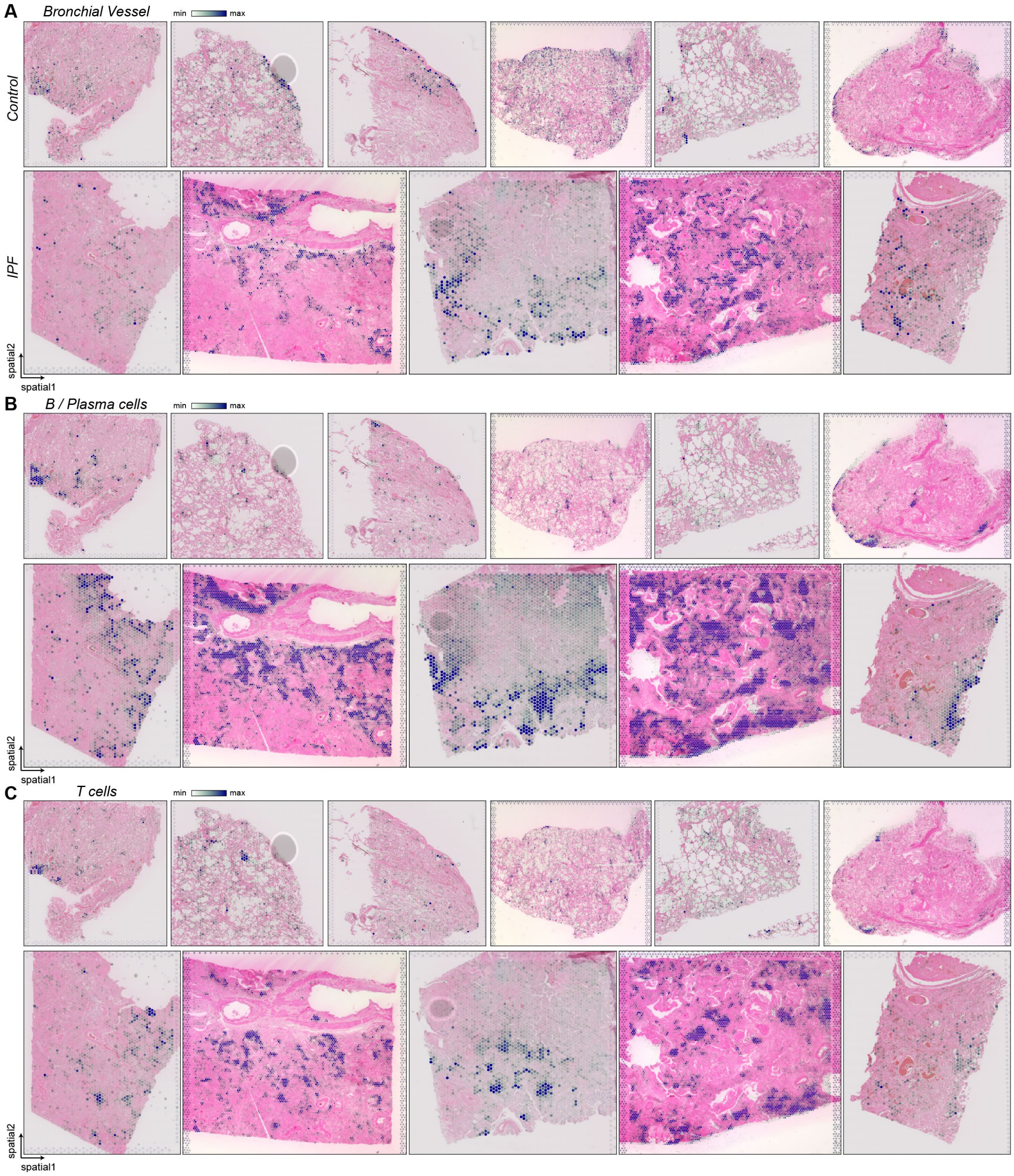

Figure S18: Localization of Immune niche contributing cell types.
(A-C) The spatial plots display the cell type abundance for indicated immune cell types across all tissue sections for **(A)** bronchial vessel cells, **(B)** B and plasma cells and **(C)** T cells.

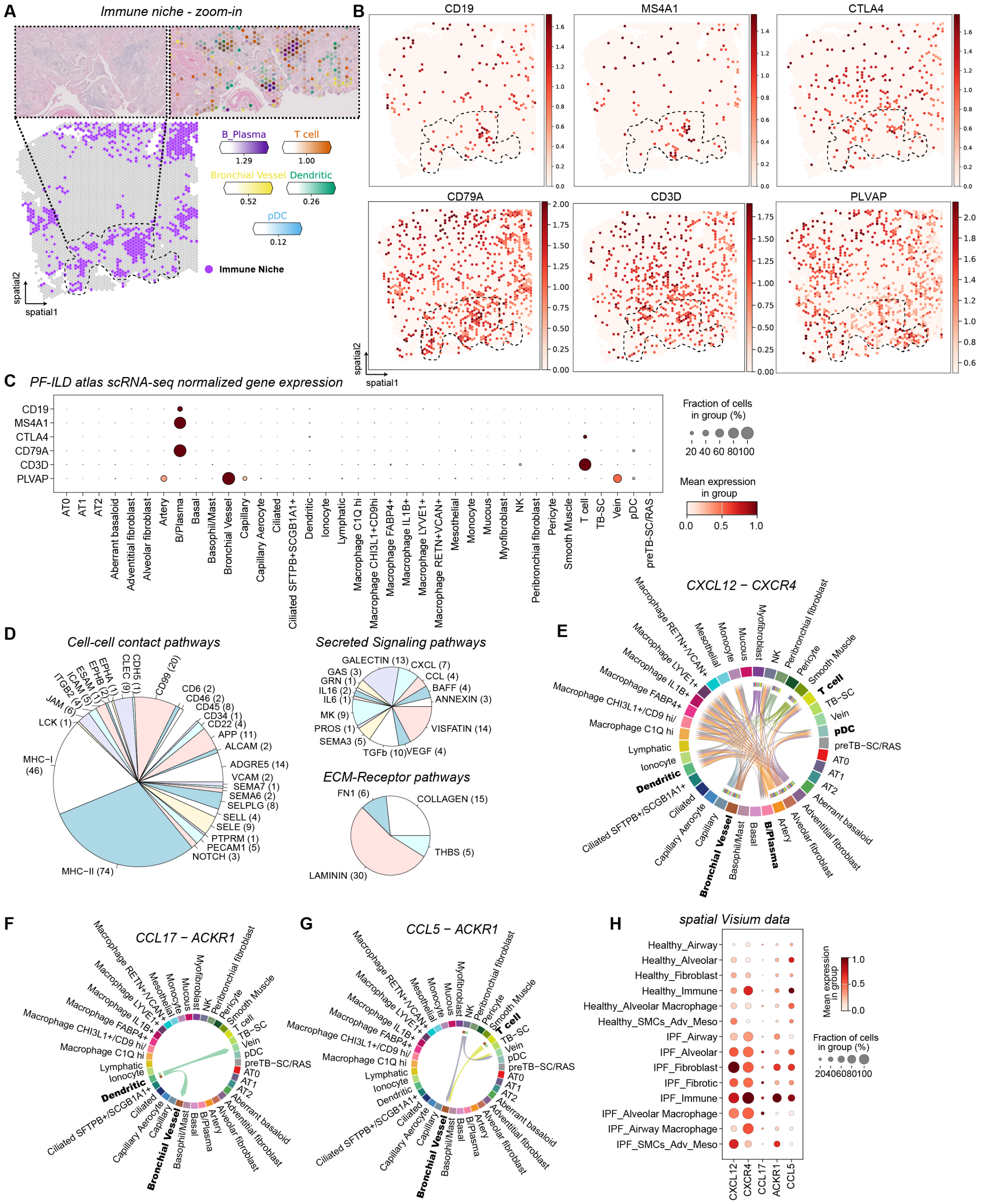

Figure S19: characterization of the immune niche.

(A-B) For one IPF tissue section, the spatial plots display **(A)** the distribution of the fibrotic niche across the slides and zoom ins of two example regions with of selected cell type abundances, as well as **(B)** indicated marker gene expression. **(C)** The dotplot displays indicated gene expression across cell types in the IPF single cell atlas. **(D)** Pie charts show the distribution of receptor-ligand pairs in the three cell communication categories and their associated signaling pathways. **(E-G)** Chord plot visualizing the interacting cell types for the ligand-receptor pair **(E)** *CXCL12-CXCR4*, **(F)** *CCL17-ACKR1* and **(G)** *CCL5-ACRK1*. **(H)** Gene expression of given receptor and ligand genes across the niches and diagnosis in the spatial Visium data.

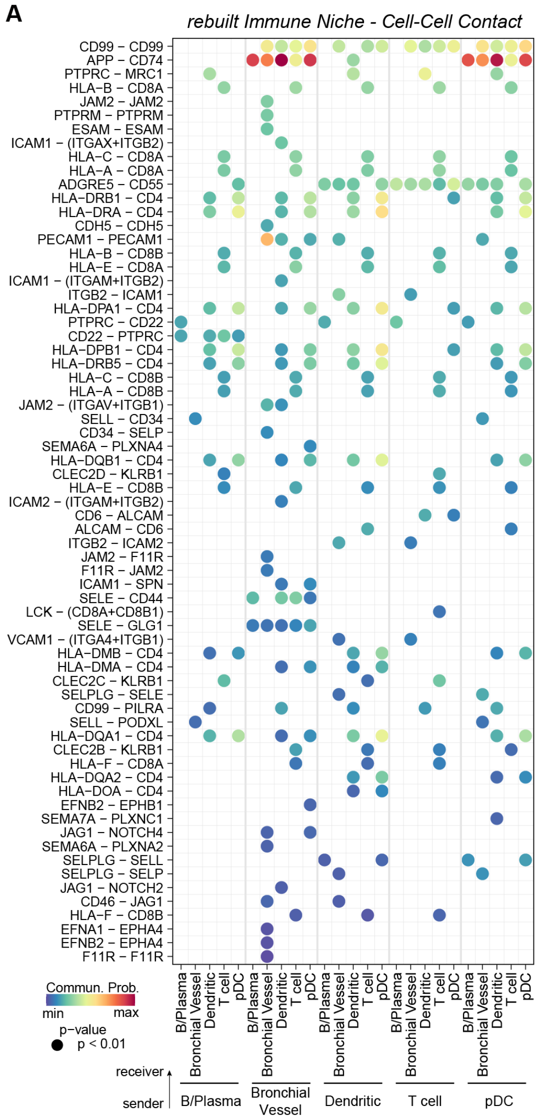

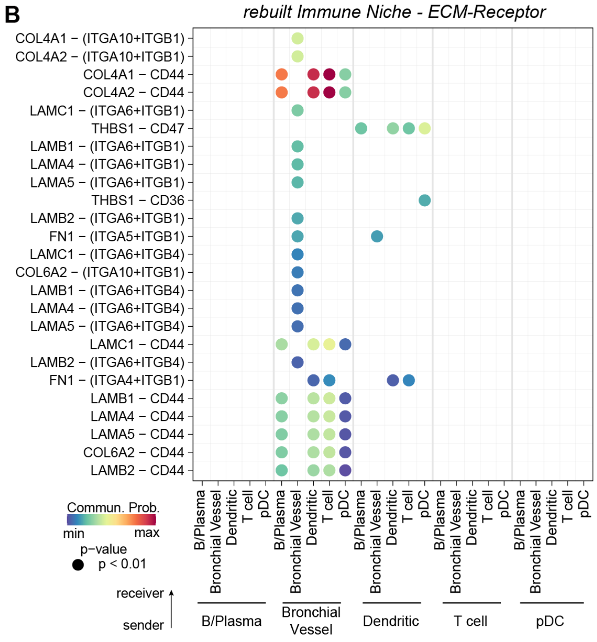

Figure S20: characterization of the immune niche.

(A-B) Heatmaps of significant ligand-receptor pairs from the **(A)** cell-cell Contact and **(B)** the ECM-receptor category within the immune niche.

Supplemental Tables

Table S1: Marker genes scRNA-seq data (cell type level).

Table S2: Baseline characteristics of human IPF and control samples used in this study

	Diagnosis	Sex	Age	Technology
ctrl_1	healthy	male	49	Visium
ctrl_2	healthy	female	32	Visium
ctrl_3	healthy	male	58	Visium
ctrl_4	healthy	female	76	Visium
IPF_1	IPF	male	51	Visium
IPF_3	IPF	male	59	Visium
IPF_2	IPF	female	32	Visium
IPF_4	IPF	male	51	Xenium
IPF_5	IPF	male	63	Xenium
IPF_6	IPF	male	54	Xenium

Table S3: Marker genes Visium data (niche level).

Table S4: Cell communication results from CellChat on the rebuilt fibrotic niche in scRNA-seq data.

Table S5: Cell communication results with from CellChat on the rebuilt airway macrophage niche in scRNA-seq data.

Table S6: Cell communication results from CellChat on the rebuilt immune niche in scRNA-seq data.

Table S7: Marker genes used to annotate cell types in the scRNA-seq PF-ILD atlas

Lymphoid cell markers:

1. T- and NK-cell markers
   - 1.1. T cell: CD3D,
   - 1.2. Cytotoxic T: CD8A, GNLY, NKG7
   - 1.3. helper T cell: CD4, RBPJ
   - 1.4. regulatory T cell: IL2RA, FOXP3, IKZF2
   - 1.5. memory/effector: CD44, SEL
   - 1.6. NK: GNLY, NKG
   - 1.7. tissue resident T: ITGAE, S1PR1, S1PR5, ITGA1, CASZ1, RBPJ, PDCD1
   - 1.8. Activated NK: XCL1, XCL2

2. CD8 markers:
   - 2.1. naïve cells: CCR7, IL7R
   - 2.2. memory cells: EOMES, CXCR3
   - 2.3. effector memory cells: FGFBP2, KLRD1
   - 2.4. activated cells: IFNG, TNF, FOS, JUN

2.5. chronically activated /exhausted cells: PDCD1, HAVCR2, CTLA4, TIGIT, LAG3

3. Plasma, dendritic and B cell markers:
   3.1. Plasma B: CD38, TNFRSF17, IGHG1, IGHG4
   3.2. B cell: CD79A, MS4A1, CD19, CD22, TCL1A, CD83
   3.3. pDC: GZMB, IGJ, ITM2C, TCF4, BCL11A, MZB1, DERL3, IL3RA, ZFAT, NRP1, IRF8, SCL15A4, BNLK, CLEC4C, LILRA4
   3.4. myeloid: FCER1G, CST3, PLAUR
   3.5. Dendritic: ZBTB46
   3.6. DC1: CLEC9A, IDO1, XCR1
   3.7. DC2: CLEC10A, FCER1A, CD1C
   3.8. DC-LAMP3: LAMP3, CCR7
   3.9. pDC: GZMB, IGJ, ITM2C, TCF4, BCL11A, MZB1, DERL3, IL3RA, ZFAT, NRP1, IRF8, SCL15A4, BNLK, CLEC4C, LILRA4
   3.10. DC4: FCGR3A, SERPINA1, LST2, AIF1, TCF72L, IFITM3, LILRB2, CSF1R, IFITM2, PECAM1, SIGLEC10, SLC7A7, LILRA2
   3.11. DC CXCL2+/CD14+/CD1C-, DC2 C1QC+, DC2 CD1A+/CD1E+
   3.12. Alveolar macrophages: FABP4, PPARG
   3.13. Dividing cells: MKI67
   3.14. Monocytes: S100A9, VCAN, IFITM2
   3.15. Dendritic cells: ZBTB46
   3.16. Macrophage: MARCO, MERTK, C1Q
   3.17. Macrophage: CHI3L1+CD9hi, Macrophage FABP4+/PDE4C+
   3.18. Macrophage IL1B+, Macrophage LYVE1+, Macrophage RETN+/VCAN+
   3.19. Monocytes: CD14+, Monocytes CD14+/IL1B, Monocytes CD16+
   3.20. Bronchial Vessel: CXCL12+, Bronchial Vessel SELE+
   3.21. Fibroblasts: COL13A1+

4. Epithelial Cells marker:
   4.1. Basal: KRT5, KRT17
   4.2. Aberrant basaloid: COL1A1, KRT17
   4.3. Mucous/Goblet: MUC5AC
   4.4. Club: SCGB3A2
   4.5. Ciliated: TPPP3
   4.6. AT1: AGER, CAV1, CAV2
   4.7. dividing: MKI67
   4.8. Ionocyte: FOXI1
   4.9. AT2: HHIP, WIF1, CA2, NNMT, SFTPC, SFTPA1,

5. Mesenchymal cell markers:
   5.1. pericyte: RGS5, MCAM

5.2. ASM: MYH11, MYL9

5.3. Alveolar fibroblast: GPC3, FGFR4, CES1

5.4. Adventitial fibroblast: PI16, C3, PLA2G2A, IGFBP6

5.5. Lipofibroblast: PLIN2, APOE

5.6. Myofibroblast: CTHRC1, COL3A1, SPON2, ASPN

5.7. Peribronchial: WIF1

5.8. dividing: MKI67

For recently described epithelial cell types we followed the respective markers(*59,60*):

- pre-TB-SC(*59*) / RAS (*60*): SCGB3A1+/SCGB1A1+/SFTPB+/SFTPC-/HHIP-
- TB-SC(*59*): SCGB3A1+/SCGB1A1-/SFTPB+/SFTPC-/HHIP-
- AT0(*59*): SCGB3A1+/SCGB1A1-/SFTPB+/SFTPC+/HHIP-
